# Recent progress of catalytic methane combustion over transition metal oxide catalysts

**DOI:** 10.3389/fchem.2022.959422

**Published:** 2022-08-08

**Authors:** Yuan Gao, Mingxin Jiang, Liuqingqing Yang, Zhuo Li, Fei-Xiang Tian, Yulian He

**Affiliations:** ^1^ UM-SJTU Joint Institute, Shanghai Jiaotong University, Shanghai, China; ^2^ State Key Laboratory of Chemical Engineering, East China University of Science and Technology, Shanghai, China; ^3^ Department of Chemical Engineering, Shanghai Jiao Tong University, Shanghai, China

**Keywords:** methane, catalytic combustion, heterogeneous catalysis, transition metal oxide, reaction mechanism

## Abstract

Methane (CH_4_) is one of the cleanest fossil fuel resources and is playing an increasingly indispensable role in our way to carbon neutrality, by providing less carbon-intensive heat and electricity worldwide. On the other hand, the atmospheric concentration of CH_4_ has raced past 1,900 ppb in 2021, almost triple its pre-industrial levels. As a greenhouse gas at least 86 times as potent as carbon dioxide (CO_2_) over 20 years, CH_4_ is becoming a major threat to the global goal of deviating Earth temperature from the +2°C scenario. Consequently, all CH_4_-powered facilities must be strictly coupled with remediation plans for unburned CH_4_ in the exhaust to avoid further exacerbating the environmental stress, among which catalytic CH_4_ combustion (CMC) is one of the most effective strategies to solve this issue. Most current CMC catalysts are noble-metal-based owing to their outstanding C–H bond activation capability, while their high cost and poor thermal stability have driven the search for alternative options, among which transition metal oxide (TMO) catalysts have attracted extensive attention due to their Earth abundance, high thermal stability, variable oxidation states, rich acidic and basic sites, etc. To date, many TMO catalysts have shown comparable catalytic performance with that of noble metals, while their fundamental reaction mechanisms are explored to a much less extent and remain to be controversial, which hinders the further optimization of the TMO catalytic systems. Therefore, in this review, we provide a systematic compilation of the recent research advances in TMO-based CMC reactions, together with their detailed reaction mechanisms. We start with introducing the scientific fundamentals of the CMC reaction itself as well as the unique and desirable features of TMOs applied in CMC, followed by a detailed introduction of four different kinetic reaction models proposed for the reactions. Next, we categorize the TMOs of interests into single and hybrid systems, summarizing their specific morphology characterization, catalytic performance, kinetic properties, with special emphasis on the reaction mechanisms and interfacial properties. Finally, we conclude the review with a summary and outlook on the TMOs for practical CMC applications. In addition, we also further prospect the enormous potentials of TMOs in producing value-added chemicals beyond combustion, such as direct partial oxidation to methanol.

## 1 Introduction

CH_4_ is the cleanest fossil fuel with the highest energy content (50–55 MJ/kg) among all sources. It emits 50–60% less CO_2_, ∼ 80% less nitrogen oxides (NO_x_), and almost negligible amount of toxic air pollutants including SO_x_, Hg, and PM 2.5 compared to that of coals (He L. et al., 2020; Zhang et al., 2021). Today, it has been well recognized that CH_4_ will play an indispensable role in the paradigm shift to a more sustainable planet. However, as the main component of natural gas, CH_4_ is the second-largest greenhouse gas after CO_2_ with a global warming potential almost 86 times that of CO_2_ over 20 years (He Y. L. et al., 2020).

Since the beginning of the industrial revolution, CH_4_ emissions have raced past 1,900 ppb in 2021, almost triple its pre-industrial levels, and contributed about 20% to the global greenhouse effect so far (Pratt and Tate, 2018; Palella et al., 2021). Specifically in China, circumstances like the incomplete combustion of fossil fuels, natural gas extraction, animal enteric fermentation, crop cultivation, agricultural residue incineration, and solid waste landfills, etc., have resulted in an annual atmospheric CH_4_ emission taking up about 60% of the total non-CO_2_ greenhouse gas emissions (Teng et al., 2019; Gong and Zeng, 2021). In this sense, the environmental benefits of CH_4_ resource itself will be largely balanced by the unburned emission, if no strict remediation plan in the exhaust stream is considered.

Catalytic combustion is one of the most effective means for CH_4_ utilization for both clean power generation and emission control (Setiawan et al., 2015; Seeburg et al., 2018). Compared to the traditional flame combustion, where high operational cost and toxic substances such as carbon monoxide (CO) and NO_x_ caused by the high combustion temperatures (>1,400°C) are almost inevitable (Stoian et al., 2021), flameless combustion of CH_4_ under the aid of a solid catalyst allows for not only a much lower operation temperature and much reduced NO_x_ emission but also a wider range of air-to-fuel ratio with a more stable and efficient combustion process outside the combustion limits (He L. et al., 2020).

At the heart of the CMC reaction lies the development of combustion catalysts, of which the catalytic performance and the cost should be properly balanced for practical implementations. To date, the most extensively explored CMC catalysts are precious metals like palladium, platinum, and rhodium, as is the case in the three-way catalytic converter for emission control in automobiles (Choya et al., 2021).

Noble metal catalysts are typically good at activating the C–H and O–O bonds to form free radicals and triggering the chain reactions, thus driving CMC to a low-temperature regime, while their high cost has never stopped the community searches for promising alternatives of cheaper price (Liu et al., 2015). Over the past decades, TMOs have received tremendous attention as one of the most promising candidates for catalyzing CMC owing to their great Earth abundance, low toxicity, and many outstanding physiochemical properties. Comparable, if not superior, performance to noble-metal-based systems has been reached in some cases such as Fe_2_O_3_, Co_3_O_4_, etc., (Li J. A. et al., 2019; He Y. L. et al., 2020; Zheng Y. F. et al., 2020; Yang et al., 2022), while with that being said, their practical uses are still limited by the low-temperature activity, resistivity against water, and sulfur tolerance in the combustion environment. Further improvement of the catalytic performance of TMO catalysts would require a well understanding of the active site structures and the corresponding reaction mechanisms, which unfortunately still remain largely controversial at the current stage, primarily due to 1) the high structural complexity of oxides themselves, involving a variety of surface acidic and basic sites; 2) the competitions between lattice oxygen and the molecular oxygen as the oxidizing sources; 3) oxygen exchange behaviors at the solid–gas interface; and 4) various adsorbed oxygen species (Toscani et al., 2019).

This review presents a systematic compilation of TMO-catalyzed CMC with special focus on the reaction mechanisms. We start with a brief introduction of the scientific fundamentals of the CMC reaction. Then, we move on to discuss the unique and desirable features of TMOs for the CMC reaction and different CH_4_ activation pathways, followed by a detailed introduction of four different kinetic reaction models. Next, we categorize the TMOs of interests into single and mixed systems, summarizing their specific morphology characterization, catalytic performance, kinetic properties, with special emphasis on the reaction mechanisms and interfacial properties. Finally, we conclude the review with a summary and outlook on the TMOs for practical CMC applications. Last but not least, we also further prospect the enormous potentials of TMOs beyond CMC, such as partial oxidation to value-added chemicals.

## 2 Reaction fundamentals

### 2.1 Catalytic CH_4_ combustion reaction

CH_4_ is an extremely stable and highly symmetrical tetrahedral structure formed by four identical C–H bonds. In CH_4_, the *sp* ^*3*^-hybridized carbon atom locates in the center of the regular tetrahedron, and four hydrogen atoms are distributed on the respective four vertices. CH_4_ possesses a high ionization potential (12.5 eV), a low electron affinity (4.4 eV), and a high C–H bond energy (434 kJ/mol), rendering both the nucleophilic and electrophilic attacks on CH_4_ extremely challenging under mild conditions (Vickers et al., 2015). The complete oxidation of CH_4_ proceeds through the following reaction (1):

$${\text{CH}}_{4}+{2\text{O}}_{2}={\text{CO}}_{2}+{2\text{H}}_{2}\text{O }\Delta {\text{H}}_{\left(298\text{K}\right)}\mathrm{=-}803\text{kJ/mol}$$ (1)

As the combustion reaction is highly exothermic, side reactions such as CH_4_–CO_2_ reforming (Eq. 2), CH_4_-steam reforming (Eq. 3), reverse water gas shift reaction (Eq. 4), partial oxidation (Eq. 5), and water gas shift reaction (Eq. 6) are almost inevitable at high temperature, as shown in the following reactions (Horn and Schlögl, 2014):

$${\text{CH}}_{4}+{\text{CO}}_{2}=2\text{CO}+{2\text{H}}_{2}\Delta {\text{H}}_{\left(298\text{K}\right)}=247\text{kJ/mol}$$ (2)

$${\text{CH}}_{4}+{\text{H}}_{2}\text{O}={3\text{H}}_{2}+\text{CO}\Delta {\text{H}}_{\left(298\text{K}\right)}=205\text{.}7\text{kJ/mol}$$ (3)

$${\text{CO}}_{2}+{\text{H}}_{2}={\text{CO }+\text{ H}}_{2}\text{O}\Delta {\text{H}}_{\left(298\text{K}\right)}=41\text{.}1\text{kJ/mol}$$ (4)

$${\text{CH}}_{4}+{1\text{/}2\text{O}}_{2}=\text{CO}+{2\text{H}}_{2}\Delta {\text{H}}_{\left(298\text{K}\right)}\mathrm{=-}36\text{kJ/mol}$$ (5)

$$\text{CO}+{\text{H}}_{2}\text{O}={\text{H}}_{2}+{\text{CO}}_{2}\Delta {\text{H}}_{\left(298\text{K}\right)}\mathrm{=-}41\text{.}9\text{kJ/mol}$$ (6)

In the 1970s, Pfefferle *et al.* proposed the process of “heterogeneous catalytic combustion” (Pfefferle and Pfefferle, 1987), where a solid catalyst can be used to combust CH_4_ in a flameless manner at a much lower reaction temperature (<500°C). Compared with traditional homogeneous flame combustion, the catalytic process owns advantages such as low light-off temperatures, reduced pollutant emission, wide operation ranges, and lower reactor requirement.

**FIGURE 1:**
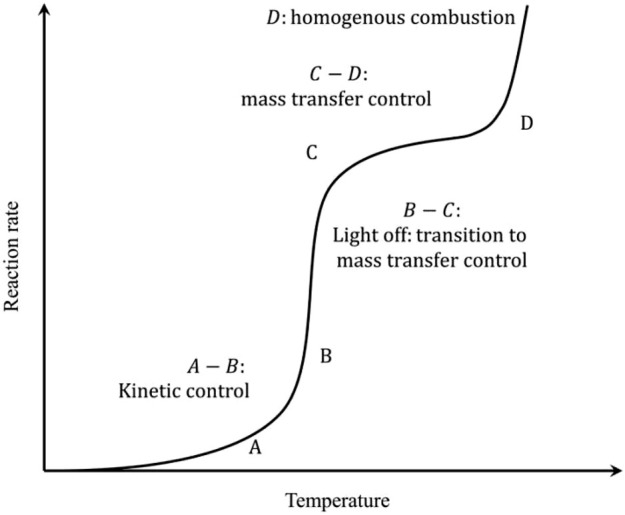
The reaction rate of CMC as a function of temperature. Reprinted with permission from He L. et al. (2020). Copyright (2020) Elsevier.

The CMC involves heterogeneous reactions, where complex processes such as mass transfer and heat transfer occur between the catalyst surface and the gas phase. Figure 1 shows the schematic relation between the reaction rate and temperature of CMC (He L. et al., 2020).

First part of the curve (A–B) is the kinetic-control zone, in which the reaction rate increases slowly with reaction temperature. At low temperatures, the reaction is limited by the intrinsic kinetics such as the dissociation rate of reactants over the catalytic surfaces. The second part of the curve (B–C) is known as the light-off regime where the reaction rate increases almost exponentially with the increase of the temperature. This is due to the drastic increase of the intrinsic reaction kinetics so that the overall reaction rates gradually transition to be controlled by the mass transfer rates. Upon further increase of the temperature (C–D), the mass transfer finally becomes the rate-limiting step, and there appears the rate plateau. The final part of the curve (D) falls under the regime of homogeneous combustion, where high temperature triggers the free radical formation and the chain reaction directly in the gas phase. The latter two high-temperature regimes impose strict requirements on the reactor design to maintain the catalyst structural stability as well as the sufficient mass and heat transfer rates. A more desirable operation window should fall nearby the light-off regime for milder reaction temperatures and sufficiently high reaction rates.

In CMC, other substances like water and sulfur are easy to get adsorbed onto catalyst surfaces under actual operation conditions; thus, the desirable catalysts for CMC should also possess a good anti-water and anti-sulfur poisoning ability beyond high low-temperature activity and thermal stability. In addition, the economic aspects should also be taken into considerations when it comes to large-scale applications.

### 2.2 Transition metal oxides

TMOs are one of the most important classes of solid catalysts that have been extensively used in a variety of important industrial processes, such as the water gas shift reaction, CO oxidation, methanol synthesis, etc., owing to their excellent thermal stability, variable valence states, and Earth abundance (Tepamatr et al., 2016; Arena et al., 2017; Huang et al., 2021). In water-containing feed, the surface of TMOs undergo acid–base reaction to form surface hydroxyl groups, which react with other reactants *via* proton or electron transfer (Yang et al., 2022). Transition metal elements could easily alternate between the low oxidation state and the high oxidation state, thus allowing for a facile release and restoration of the lattice oxygen *via* a redox cycle (Chen et al., 2015). Such redox and acid–base properties have rendered TMOs to be one of the most promising candidates for CMC reactions.

TMOs like Co_3_O_4_, Fe_2_O_3_, MnO_2_, etc., have shown catalytic activities comparable to that of noble metal-based systems in CMC (Pu et al., 2017; He Y. L. et al., 2020; Yang et al., 2021), while the performance can be largely affected by preparation method, carrier, doping, and experimental conditions (such as the presence of water vapor and sulfur). The most common preparation methods of TMOs include thermal decomposition, precipitation, combustion synthesis, hydrothermal method, dipping, and sol–gel method (Tang et al., 2008). The different preparation methods can lead to diverse structural properties such as particle size, dispersion, surface area, morphology, crystal plane, metal valence states, defects, and reactive oxygen species. Such variances in the TMO crystal structures may result in drastic differences in the expression of catalytic activity, as was found in Co_3_O_4_, Fe_2_O_3_, IrO_2_, etc., where only certain crystal facets are reactive for CMC reactions (Liang et al., 2017; He L. et al., 2020).

The main exposed crystal planes of TMOs with different morphologies have a significant effect on the catalytic activity of CMC. For example, the (111) and (112) crystal planes of Co_3_O_4_ and the (110) crystal planes of Fe_2_O_3_ are considered to be the best catalytic activity for CMC (Hu et al., 2008; He L. et al., 2020). In addition, the structure dimensionality also induces different catalytic properties due to quantum confinement and geometric effect, as was seen in Co_3_O_4_ where the 2D nanosheets and 3D nanoflowers possess drastically different catalytic performances (Jia Y. C. et al., 2016).

TMO-based catalysts are generally supported in CMC for improved particle dispersion, increased specific surface area, and enhanced catalytic performance. Both inactive and active supports are used to disperse TMOs in CMC. For example, Al_2_O_3_, as an inactive support, is an acidic oxide capable of adsorbing CH_4_ through acid–base pairing (Wang and Lin, 2004; Choya et al., 2018a). Active supports, such as CeO_2_, can directly participate in the CMC reaction through its excellent oxygen storage capacity (Pecchi et al., 2005; Li Y. X. et al., 2009).

Doping TMOs with an impurity element is also an effective strategy to improve their catalytic performance in CMC by inducing a great deal of lattice defects, acidic and basic sites, oxygen vacancies, synergistic effect, etc., (Li J. H. et al., 2009). The increased amount of oxygen vacancies can not only improve the mobility of bulk oxygen in the lattice but also creates additional adsorption sites to promote the molecular adsorption from the gas phase, reducing the activation barrier (Wang H. W. et al., 2019). The dopants in TMOs include metal and non-metal elements. Elements like Ca and Mg are often used as promoters, while Ce and Mn are doped to obtain oxygen vacancies (Pecchi et al., 2011; Chen Y. L. et al., 2021). N is a commonly used dopant in non-metal doping, which can create more lattice distortions and further increase active oxygen species (Buchneva et al., 2012; Li et al., 2018).

One of the most serious issues for TMO catalysts in CMC is their resistance to water poisoning. Most polar facets of TMOs are prone to dissociatively adsorb moisture through an acid–base reaction and form rich surface hydroxyl groups, while this process is highly reversible and self-dehydration can occur at elevated temperatures (He Y. L. et al., 2020). Water vapor on the surface of TMOs reacts with chemisorbed oxygen, which hinder the delivery of reactive oxygen species to active sites. Moreover, the formation of hydroxyl groups hinders the desorption of H_2_O and CO_2_ from the surface of TMOs (He L. et al., 2020). Water vapor poisoning is generally reversible owing to the competitive adsorption of water molecules with reactants. Some TMOs possess inherent water tolerant stability, such as NiO, owing to the modification of water for NiO. Tuning the structure and surface topography of the TMO catalysts is effective in obtaining resistance to water vapor (Liu et al., 2017; Xu et al., 2017). Hydrophobic modification of TMOs or their supports may also be an effective means to improve the water resistance and catalytic activity of catalysts (Kuo et al., 2014).

Sulfur poisoning is another serious issue for TMO catalysts in CMC. Natural gas contains a substantial amount of sulfur element, which can be oxidized into sulfur dioxide (SO_2_) and get adsorbed onto the catalyst surface of the catalyst; subsequent oxidation to sulfate could also occur and thus cause serious sulfur poisoning. Some TMOs, such as Cr_2_O_3_, inherently have excellent sulfur resistance due to its low affinity to acid gases (Ordóñez et al., 2008). Sacrificial component such as Mn-based oxide can be used to guarantee the sulfur resistance of the CMC catalyst (Zhong et al., 2019). Similarly, supports such as Al_2_O_3_ can react with SO_2_ to form sulfates, which protects active sites. But it is unfavorable for the recovery of TMO catalysts. In fuel-rich conditions, an elevated temperature (>500°C) is an effective means of recovery for sulfur-poisoned TMO catalysts (Gremminger et al., 2017).

In addition, carbon deposition can also cause serious catalyst deactivation in CMC, as commonly seen in carbonaceous reactions. Note that the effect can be remediated through carefully controlled reaction conditions, such as adjusting the air-to-fuel ratio, the space velocity, and the reaction temperature. In lean combustion scenario where the oxygen content is in excessive amount, the CH_n_ species formed from CH_4_ dissociation can be rapidly oxidized by dioxygen to form CO_2_, thus avoiding coke formation. While if a rich fuel mixture is used, the insufficient oxygen content can result in the formation of coke-like carbon (Tao et al., 2015). Under the circumstance of oxygen source coming from lattice oxygen rather than the molecular oxygen in the gas phase, the CH_4_ to TMO catalyst feed ratio should be instead taken in consideration correspondingly. When the lattice oxygen in the TMO catalysts is insufficient to react with CH_4_, CH_4_ will decompose and then form carbon deposition (Wu and Ku, 2018). Another factor that can induce the catalyst deactivation is the space velocity, a small space velocity of the feed can lead to serious carbon deposition due to the long residence time between CH_n_ species and the catalysts (He Y. L. et al., 2020). Furthermore, carbon deposition may also result from the decomposition of CH_4_ at high temperatures. Promoting coke oxidation can prolong the lifetime of TMO catalysts. TMOs with high oxygen storage capacity such as CeO_2_ can remove coke deposition through the release of lattice oxygen (Wang Y. N. et al., 2022). Therefore, sufficient oxygen content, reasonably short residence time, and low reaction temperature are necessary to reduce carbon deposition in CMC. For TMOs, in particular, a high oxygen storage capacity will be helpful to prevent coke formation.

### 2.3 Reaction mechanisms

The reaction mechanisms proposed for CMC over many TMOs remain to be controversial, primarily due to the high structural complexity, different oxygen sources, oxygen-exchange behaviors, etc. Such knowledge gaps hinder us from understanding the precise active site structures and the reaction pathways from a molecular level and consequently the optimization of TMO catalysts for CMC. To this end, the existing mechanistic studies of CMC over TMOs are summarized in this section.

#### 2.3.1 C–H and O–O activation

Dissociation of the first C–H bond is the most critical step of CH_4_ activation and the following CMC reactions (Feyel et al., 2006). Fu *et al.* studied different C–H activation mechanisms over various TMOs using cluster model calculations. It was found that H abstraction is the primary working mechanism for the activation of CH_4_ on most TMOs such as Cr_3_O_9_ and Mo_3_O_9_, following the hydrogen atom transfer (HAT) route. H abstraction is a single-electron transfer process forming free radicals *via* the homolytic cleavage of the C–H bond, in which the H atom attacks the terminal oxygen atom from both *trans* and *cis* directions. In terms of activation energy, the H abstraction process in *trans* on the terminal oxygen is more favorable than that from the *cis* directions (Fu et al., 2006). Li *et al.* believed that there is also a proton-coupled electron transfer (PCET) process for the activation of C–H bonds on TMOs, that is, transition metal oxide ions and O^2−^ as a Lewis acid–base pair are served as catalytic active sites. In the process, a proton is abstracted from CH_4_ by the Lewis basic O^2−^, while the methyl anion is transferred to the Lewis-acidic metal center (Li et al., 2016). Thus, the activation of CH_4_ on TMOs is usually closely related to the acidity and alkalinity of the surface.

In addition to CH_4_ activation, the molecular oxygen also plays a significant role in governing the catalytic performance in CMC. The composition and concentration of reactive oxygen species significantly affect the CMC reaction over TMOs. In CMC, there are primarily two types of oxygen species involved in the TMOs, including surface-adsorbed oxygen and lattice oxygen. According to Eq. 7, the dissociative adsorption of molecular oxygen will result in the formation of a series of different adsorbed oxygen species with electrophilic characters, in the sequence of superoxide O_2_^−^, peroxide O_2_^2−^, and charged atomic species O^−^. The reaction ultimately leads to the incorporation of nucleophilic O^2−^ into the lattice to form lattice oxygen (Wang Y. Q. et al., 2022). It is precisely because of the different activation forms of C–H bonds and oxygen molecules that the mechanism of CMC is such diverse

$$\begin{array}{l} {\text{O}}_{2}\left(\text{g}\right)\overset{\left|*\right|}{↔}{\text{O}}_{2}\left(\text{s}\right)\overset{+{\text{e}}^{-}}{↔}{\text{O}}_{2}^{-}\left(\text{s}\right)\overset{+{\text{e}}^{-}}{↔}{\text{O}}_{2}^{\mathrm{2-}}\left(\text{s}\right)\overset{\left|*\right|}{↔}{2\text{O}}_{2}^{-}\left(\text{s}\right)\overset{+2{\text{e}}^{-}+2{\text{V}}_{\text{0}}}{↔}{2\text{O}}^{2-}\left(1\right)\\ {}*-\text{a free adsorption site; }\\ \text{g}-\text{gas surface; l}-\text{lattice} \end{array}$$ (7)

#### 2.3.2 Kinetic models

**FIGURE 2:**
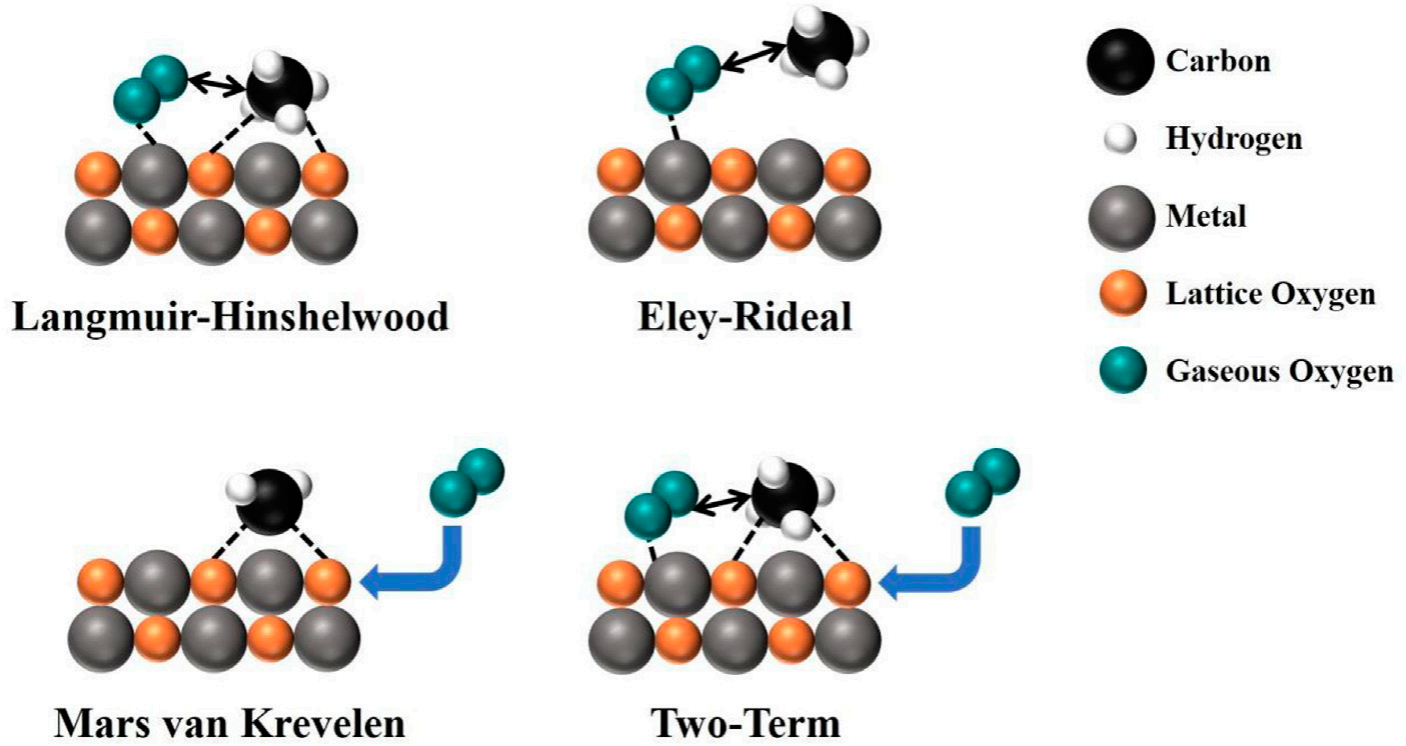
The reaction mechanism diagram of CMC.

The various forms of C–H and O–O activation mechanisms undoubtedly result in a complex reaction network for TMO-catalyzed CMC systems. According to the participation forms of different oxygen species, the proposed reaction mechanisms of the CMC currently include the following four mechanisms (Figure 2). Langmuir–Hinshelwood (L–H) mechanism and the Eley–Rideal (E–R) mechanism are dominated by surface-adsorbed oxygen, while the Mars van Krevelen (MvK) mechanism is dominated by lattice oxygen, and the two-term (T-T) mechanism is cooperatively controlled by the L–H mechanism and the MvK mechanism. The rate equations consistent with these mechanisms are as follows:

$$r_{LH}=k_{C}\frac{K_{C}P_{C}\cdot K_{O}P_{O}}{\left(1+K_{C}P_{C}\right)\left(1+K_{O}P_{O}\right)}$$ (8)

$$r_{ER}=k_{O}\frac{K_{O}P_{O}\cdot P_{C}}{\left(1+K_{O}P_{O}\right)}$$ (9)

$$r_{MvK}=\frac{k_{C}P_{C}\cdot k_{O}P_{O}}{vk_{C}P_{C}+k_{O}P_{O}}$$ (10)

**L–H mechanism**. It is believed that the adsorption activation energy of O_2_ on the catalyst surface is much smaller than that of CH_4_, and O_2_ is preferentially adsorbed on the catalyst surface to form adsorbed oxygen. Meanwhile, adsorbed oxygen species are easier to combine with adsorbed CH_4_ than O_2_ due to their electrophilic attack on CH_4_, so that the hydrogen in CH_4_ is dissociated, destroying the stable structure of CH_4_ and generating active methyl radicals to promote the oxidation of CH_4_. The L–H mechanism is mainly found on noble metals and their supported oxide systems (Becker et al., 2011). Trimm *et al.* studied methane oxidation over the Pt/Al_2_O_3_ porous catalyst and found that the rate equation derived by experimental results was best fit with the L–H mechanism (Trimm and Lam, 1980).

**E–R mechanism**. In the E–R mechanism, the gaseous O_2_ is first adsorbed on the metal atoms of TMOs and then further activated to form surface-adsorbed oxygen, which reacts with gaseous CH_4_ to form active methyl species, finally oxidized to CO_2_ (Belessi et al., 2001). Veldsink *et al.* investigated the reaction rate of CMC over a commercially available CuO-γ-A1_2_O_3_ catalyst and derived a kinetic rate equation from all experimental data. The rate equation is in good accordance with the E–R mechanism, in which catalyst with adsorption of O_2_, CO_2_, and H_2_O, but does not adsorb CH_4_ (Veldsink et al., 1995).

**MvK mechanism**. MvK is a redox mechanism where the catalyst will be reduced by CH_4_ at first, and then the active centers will be reoxidized by molecular oxygen uptakes. It includes surface oxygen reaction and lattice oxygen migration, in which the reaction pathway can be divided into the following steps: the surface dissociative adsorption of gaseous CH_4_ onto the active site of the catalyst to form adsorbed alkyl and hydroxyl groups, and the reaction spills over to form CO_2_ and H_2_O products taking the lattice oxygen as the oxidizing source. After product desorption, the active centers will be reduced with a lot of formation of lattice oxygen vacancies, which can be replenished by either lattice oxygen diffusion from the bulk phase or molecular oxygen adsorption from the gas phase. This reaction mechanism is found for several TMOs like Fe_2_O_3_ and Co_3_O_4_ (Zasada et al., 2017; He L. et al., 2020).

**T-T mechanism**. Due to the complex nature of CMC, there are some cases where describing the reaction process on the catalyst by a single mechanism is rather challenging, and then the T-T mechanism is proposed, that is, both surface adsorption oxygen and lattice oxygen participate in the reaction simultaneously (Han et al., 2008). Gaseous oxygen adsorbs on metal active sites and then dissociates to form surface-active oxygen species. It oxidizes CH_4_ together with lattice oxygen species that migrate from the bulk to the surface, and the lattice oxygen vacancies will be replenished by gaseous oxygen, resulting in some more suitable explanation of reaction mechanisms in CMC over TMOs (Wang et al., 2018).

Although in some cases that an explicit reaction model can be interpreted to well describe the reaction kinetics of TMO-catalyzed CMC reactions, such as Fe_2_O_3_, Co_3_O_4_, etc., the reaction mechanism may vary based on the structure of TMOs or the reaction conditions. For example, Zasada *et al.* systematically examined CMC over Co_3_O_4_ nanocubes, revealing that the content of oxygen vacancies significantly affects the reaction mechanism. In the low-temperature range (300–450°C), CH_4_ is primarily activated by monatomic oxygen species on the catalyst, in which facile decarboxylation and dehydroxylation leave the catalyst surface stoichiometric, thus following the L–H mechanism. From 450 to 650°C, the intrafacial dehydroxylation and decarboxylation of the catalyst accelerated the formation of oxygen vacancies, making the catalyst surface slightly reduced. Then, oxygen vacancies are virtually refilled by O_2_, which is in accordance with the coexistence of L–H and MvK mechanisms. Above 650°C, oxygen vacancies formed from the bulk Co_3_O_4_, so causing the active involvement of the lattice oxygen following the MvK mechanism. In this case, the varying reaction conditions change the catalyst redox states, in which the number of oxygen vacancies leads to a variety of reaction mechanisms (Zasada et al., 2017).

**FIGURE 3:**
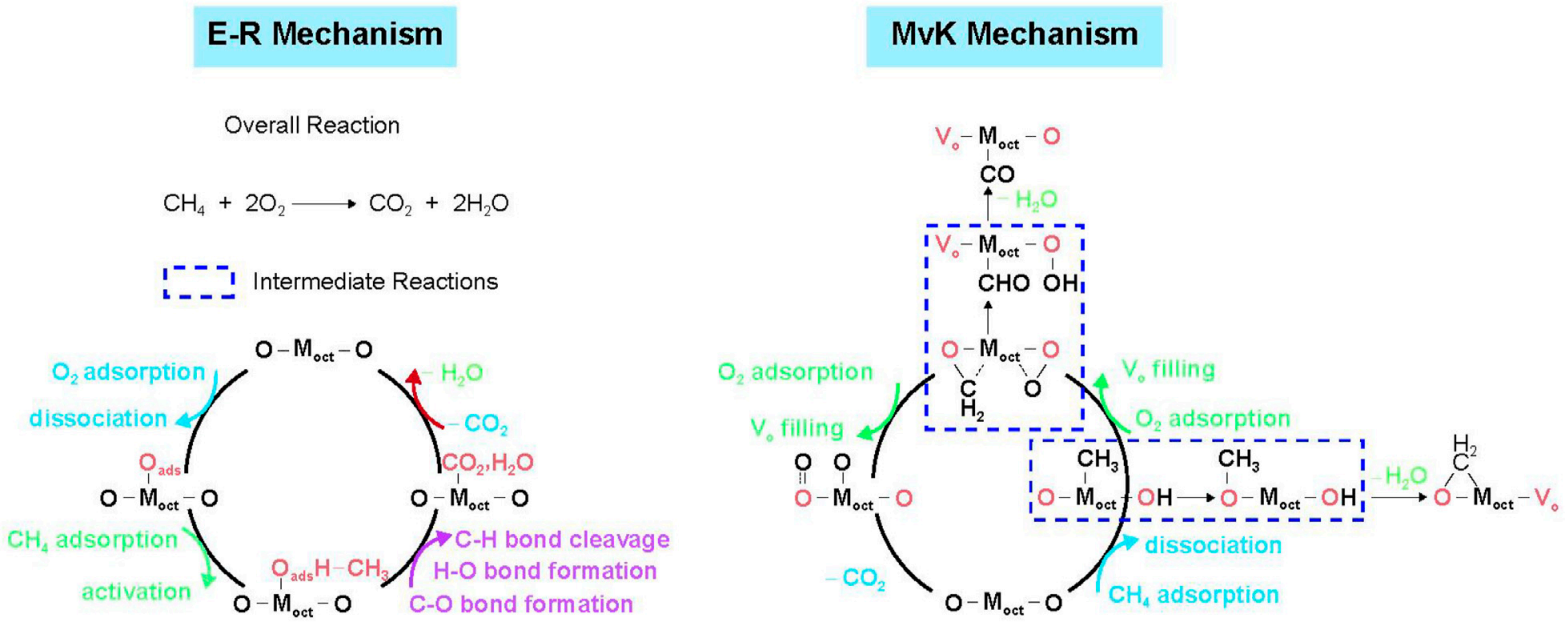
The proposed reaction pathways based on the E–R mechanism for Ni-poor spinel oxides and MvK mechanism for Ni-rich spinel oxides. Cited from Wang H. W. et al. (2019).

The mechanism of CMC may also be significantly altered by doping or substitution of other transition metal ions with different contents into transition metal oxides. Wang *et al.* demonstrated that the catalytic behavior can be regulated through the substitution of Co by Ni in spinel ZnNi_x_Co_2-x_O_4_ oxides (Figure 3). In addition, the difference of catalytic behavior can be explained by the interaction between the O *p*-band center and metal *d*-band center. When the metal *d*-band center exhibits a higher position relative to the O *p*-band center in Ni-poor ZnNi_x_Co_2-x_O_4_ spinel oxides, the catalyst shows a greater metal character, following the E–R mechanism, among which the first C–H bond cleavage and the H_2_O desorption are considered to be the rate-determining steps. On the contrary, Ni-rich spinel oxides with the higher O *p*-band center compared with the metal *d*-band center show greater oxygen character, in accordance with the MvK mechanism, in which the multiple lattice oxygen involved steps are crucial for the entire methane oxidation (Wang T. et al., 2019).

The mechanism of CMC may also be greatly affected by the reaction temperature. He *et al.* investigated CMC over α-Fe_2_O_3_ *via* a combined experimental and theoretical study and found that C^16^O_2_ formed first, followed by H_2_^16^O, C^16,18^O_2_, H_2_^18^O, and finally C^18^O_2_, where ^18^O_2_ was only used in the gaseous molecular oxygen. The numerical analysis of the mass balance revealed that the ratio ^16^O/^18^O > 1 at T < 385°C, then ^16^O/^18^O ≈ 1 at T ≈ 385°C, and finally ^16^O/^18^O < 1 at T > 385°C. These indicate that, below 385°C, the lattice oxygen plays a predominant active role in CMC reaction, in accordance with the MvK mechanism. However, above 385°C, the mechanism becomes more complex and difficult to analyze (He Y. L. et al., 2020).

## 3 Transition metal oxides for catalytic CH_4_ combustion

In the application of the CMC, the TMOs such as Co_3_O_4_, NiO, MnO_2_, Cr_2_O_3_, Fe_2_O_3_, CeO_2_, CuO, and their binary complex oxides have been widely studied. Therefore, the characteristics of these TMOs and the mechanisms of the CMC over these catalysts are comprehensively reviewed in the following sections in order to provide a general guideline about the construction of well-performed catalytic systems for the CMC. The catalytic performances of different TMOs for CMC are shown in Table 1.

**TABLE 1:** The catalytic performances of different TMOs for CMC.

TMOs	Preparation method	Surface area (m^2^ g^−1^)	Light-off temperature (°C)	E_a_ (kJ mol^−1^)	Feed composition and GHSV/WHSV (gas/weight hourly space velocity)	Stability	Reaction mechanism	References
Co_3_O_4_	basic precipitation	14	T_50_ = 330	74 ± 2	1% CH_4_, 10% O_2_ and 89% N_2_ GHSV: 60,000 h^−1^	anti-H_2_O: good (reversible deactivation) anti-SO_2_: none	MvK	Choya et al. (2022)
Co-In-0.2 oxide	a designed precipitation	57.3	T_10_ = 265	82.2	1% CH_4_, 10% O_2_, N_2_ (balance); GHSV = 48,000 ml g^−1^ h^−1^	anti-H_2_O: excellent (fully restored) anti-SO_2_:: none	none	Zheng Y. et al. (2020)
			T_99_ = 395					
N-Co_3_O_4_-110	a facial N_2_ plasma engraving	52.3	T_50_ = 342	73.0	2 vol. % CH_4_, 20 vol. % O_2_, 5 vol. % H_2_O, Ar gas; WHSV = 46,800 ml g^−1^ h^−1^	anti-H_2_O: good (reduced by about 3%) anti-SO_2_:: none	May be MvK	Yu et al. (2020)
			T_90_ = 412					
Co_3_O_4_/Ce_0.75_Zr_0.25_	solution combustion synthesis	none	T_50_ = 217 (with electric field)	35.8 (with electric field)	[CH_4_] = 0.2%, [O_2_] = 10%, N_2_ (balance gas); GHSV = 30,000 h^−1^	none	MvK	Li K. et al. (2019)
Mesoporous Co_3_O_4_	Calcining	136.2	T_10_ = 220	none	1% CH_4_, 20% O_2_; space velocity = 18,000 ml g^−1^ h^−1^	none	E-R	Han et al. (2016)
			T_50_ = 270					
Zr-Doped NiO	homogeneous co-precipitation strategy	142	T_90_ = 380	55.27	1 vol% CH_4_, 10 vol% O_2_, balanced N_2_ GHSV: 30,000 ml g^−1^ h^−1^	anti-H_2_O: good anti-SO_2_: none	MvK	Wang et al. (2021)
Ni-Cu mixed oxides	co-precipitation method	147	T_50_ = 370	89	1 vol% CH_4_, 5 vol% O_2_ and N_2_ GHSV: 50 000 ml g^−1^ h^−1^	anti-H_2_O: good anti-SO_2_: none	MvK	Fan et al. (2022)
			T_90_ = 410					
Mn-Ce-RP	redox-precipitation method	none	T_50_ = 446°C	none	CH_4_(1%)/O_2_(10%)/N_2_ WSHV = 30,000 ml/(g*h)	anti-SO_2_: excellent anti-H_2_O: none	MvK	Zhong et al. (2019)
nanocubic MnO_2_	a hydrothermal process	78	T_50_ = 293	none	none	none	L- H	Zhang et al. (2019)
			T_90_ = 350					
α-MnO_2_	a hydrothermal	92.9	T_50_ = 356	none	0.1% CH_4_ in air WHSV = 90 L g^−1^ h^−1^	none	MvK	Jia et al. (2019)
			T_90_ = 463					
MnO_2_/ZrO_2_	one-pot hydrothermal	none	T_50_ = 340	none	1,000 ppm CH_4_, 10% O_2_, and N_2_ (balance) WHSV = 45 L g^−1^ h^−1^	anti-H_2_O: good anti-SO_2_: good	none	Jia et al. (2018)
Nano-ZnCr_2_O_4_ spinel oxides	a ethylene glycol-mediated solvothermal	96.2	T_10_ = 300	144.8	2 vol% CH_4_, 20 vol% O_2_, 78 vol% N_2_ GHSV = 78,000 h^−1^	none	L-H	Huang et al. (2019)
			T_90_ = 400					
Sn-Cr binary oxide	a co-current co-precipitation	133	T_10_ = 320	none	1.0% CH_4_ in air; space velocity = 20,000 h^−1^	none	MvK	Zhu et al. (2003)
			T_50_ = 400					
			T_90_ = 490					
Fe and Cr-based oxides	the citrate sol–gel	82	T_450_°C = 79%	none	347 ppm CH_4_+5.1 ppm SO_2_	anti-H_2_O: good anti-SO_2_: excellent	none	García-Vázquez et al. (2020)
Fe_60_Cr_40_			T_550_°C = 97%					
Bulk α-Fe_2_O_3_	precipitation	none	T_50_ = 461	99.9	2000 ppm V CH_4_ in air	none	MvK (100–500°C)	Paredes et al. (2004)
Nano sheetα-Fe_2_O_3_	hard-templating	none	T_10_ = 230	17.60	5% CH_4_ and 20% O_2_ balanced with 75% Ar; WHSV = 10 000 ml g^−1^ h^−1^	none	MvK (below 400°C)	He Y. L. et al. (2020)
			T_50_ = 394					
NiO/CeO_2_	Deposition precipitation	none	T_50_ = 465	69.4 ± 4.0	20 ml/min 10% CH_4_/Ar and 10 ml/min pure O_2_ WHSV = 18 000 ml g^−1^ h^−1^	anti-H_2_O: good anti-SO_2_: none	MvK	Zhang et al. (2018)
MnCeO_x_ (Mn-to-Ce ratio of 1:3)	redox	none	T_50_ = 475	113	CH_4_/O_2_/He mixture (concentration: 1/4/95); Space velocity = 30,000 h^−1^	none	MvK	Palella et al. (2021)
			T_100_ = 700					
Ce_0.9_Zr_0.06_Sc_0.04_O_1.98_	citrate complexation	none	T_50_ = 700	124.3	1 vol% CH_4_, 8 vol% O_2_ and 91 vol% N_2_	none	MvK	Toscani et al. (2019)

### 3.1 Co_3_O_4_-based catalysts

#### 3.1.1 Single Co_3_O_4_

Co_3_O_4_ is a p-type semiconducting metal oxide with a typical spinel structure, and it has an array based on a cubic close-packed oxide ion whose lattice parameter is a = 0.811 nm with the space group of Fd3m. In the crystal structure of Co_3_O_4_, Co^2+^ occupies the tetrahedral coordination and Co^3+^ occupies the octahedral coordination, respectively, with a mean cobalt oxidation state of +2.67. Co_3_O_4_ is considered to be one of the most effective co-oxidation catalysts due to the unfilled 3*d* orbital of Co, the weak Co–O bond strength, a high cycle frequency of redox, and a low barrier of oxygen vacancy. Owing to its unique physical and chemical properties, it has a wide range of applications in sensors, magnetic materials, lithium-ion batteries, and solar cells (Sanchis et al., 2021). In CMC, Co_3_O_4_ has attracted the attention of many researchers due to its high stability, variable valence, and excellent catalytic performance. Paredes *et al.* found the activity of different bulk TMOs catalysts in methane combustion in the following order: Co_3_O_4_ > Mn_2_O_3_> Cr_2_O_3_> CuO > NiO (Paredes et al., 2009). Therefore, Co_3_O_4_ is considered to be one of the best candidates for CH_4_ combustion catalysts among all TMOs and a promising alternative for noble metal combustion catalysts.

According to the previous literature, the common methods for preparing Co_3_O_4_ include precipitation, sol–gel method, hydrothermal synthesis, impregnation, thermal decomposition, and solid phase reaction. Choya *et al.* prepared several bulk Co_3_O_4_ catalysts by various synthesis methodologies, among which the solution combustion synthesis route, the basic grinding route, the calcination of the cobalt hydroxycarbonate route, and the precipitation with the sodium carbonate route showed better textural properties than the commercial catalyst due to the higher presence of Co^3+^ on their surface and further resulted in abundant lattice oxygen species. The catalysts exhibited higher catalytic activities due to their favored mobility of lattice oxygen species (Choya et al., 2022). Wang *et al.* prepared a series of Co_3_O_4_/γ-Al_2_O_3_ catalysts by a combination of incipient wetness impregnation (IWI) and subsequent combustion synthesis (CS) method. Results revealed that the CS method exhibited higher catalytic activity than the catalysts prepared by the IWI method, attributing to the higher surface area, lower Co_3_O_4_ crystallization, better dispersion, more surface Co^3+^, as well as easier and faster redox cycle between Co^2+^ and Co^3+^ (Wang et al., 2015).

Studies have shown that the catalytic behavior of Co_3_O_4_ catalysts strongly depends on its morphology, structure, and crystal planes, which endow them with different electron transfer abilities and different amounts of active sites, promoting catalytic activity and selectivity. The morphological characteristics can be regulated by the preparation method, such as spheres, nanorods, nanowires, nanobelts, and nanosheets.

Wang *et al.* prepared Co_3_O_4_ with the shape of nanosheets and nanospheres by the hydrothermal method in media of ethylene glycol and water, respectively. The concentration of ethylene glycol and the hydrothermal temperature significantly influenced the size and shape of the Co_3_O_4_, which showed that the Co_3_O_4_ nanosheets exhibited slightly higher catalytic performance than the Co_3_O_4_ nanoparticle, as more active oxygen species were found in the former (Wang et al., 2017). Chen *et al.* controllably synthesized Co_3_O_4_ nanocrystals with different morphologies (flower, hexagonal plate, hexagonal sheet, and cube), which showed that the properties of Co_3_O_4_ were closely related to the morphology. Compared to cubical Co_3_O_4_ with the (100) plane, the flower-like, hexagonal plate-like, and hexagonal sheet-like Co_3_O_4_ catalysts were more active, which may be due to more exposed (111) planes (Chen et al., 2016).

In addition, the spatial structures of TMOs also play an important role in the catalytic activity. Sun *et al.* prepared Co_3_O_4_ catalysts with different spatial structures, such as 0D (nanoparticles), 1D (nanorods), 2D (nanoplates), and 3D (mesoporous and microporous) structures. Among them, 2D structures (nanoplates) have the best catalytic activity, which is contributed to the high refractive index of the exposed (112) crystal planes and the role of surface-active species (such as surface-adsorbed oxygen and Co^2+^) in the catalytic reaction (Sun et al., 2016).

The use of supports can effectively improve the dispersion and prevent the particle agglomeration caused by the sintering of the Co_3_O_4_ catalyst, thereby improving the catalytic activity of the catalyst. Feng *et al.* deposited Co_3_O_4_ on the SmMn_2_O_5_ (SMO) support to prepare Co/SMO composite catalysts and evaluated the performance of Co/SMO catalysts in oxygen-enriched environments. The results showed that the Co/SMO-50% catalyst had high catalytic activity and strong durability. The strong interaction between Co_3_O_4_ and SmMn_2_O_5_ played a key role in dispersing and stabilizing Co_3_O_4_ by preventing catalyst sintering. Highly dispersed Co_3_O_4_ formed more surface lattice oxygen-oxidized CH_4_, leading to the transformation from Co^3+^ to Co^2+^, along with the formation of an oxygen vacancy, which could be compensated by the gaseous oxygen, so following the MvK mechanism (Feng et al., 2018). Dou *et al.* obtained Co_3_O_4_/CeO_2_ catalysts by supporting Co_3_O_4_ nanoparticles on CeO_2_ nanorods by the deposition precipitation method. The results showed that complete oxidation of CH_4_ on Co_3_O_4_/CeO_2_ (43.9 kJ/mol) was obviously lower than pure CeO_2_ (95.1 kJ/mol) and pure Co_3_O_4_ (89.7 kJ/mol). Co_3_O_4_/CeO_2_ showed synergistic effect, in which oxygen vacancies on the surface of CeO_2_ were active centers for activating molecular oxygen in the oxidation reaction, thus promoting the improvement of catalytic activity (Dou et al., 2018).

#### 3.1.2 Doped Co_3_O_4_

The element doping has been considered as an effective way to adjust the surface and electronic structures of nanomaterials. Some studies show that introducing oxygen vacancies will have a huge effect on the oxidation reaction. In the element-doped catalyst, Co_3_O_4_, as the reactive site, actively participates in CMC, while the doping of other elements often creates more oxygen vacancies to promote the rapid migration of lattice oxygen (Rodríguez-Fernández et al., 2019). Zheng *et al.* believed that Co^2+^ was active species in Co_3_O_4_. Thus, they prepared Co-In-x oxide *via* a designed precipitation method by taking N-butylamine as the precipitant. The Co–In–O solid solution phase exhibited a superior activity by doping an appropriate amount of In^3+^ to replace the Co^3+^ site of octahedral position, increasing the proportion of active species Co^2+^, giving rise to abundant active oxygen species, improving reducibility, and optimizing surface acidity. Moreover, Co–In-x oxide also demonstrated excellent stability and water resistance, indicating that the doping of In^3+^ was beneficial to maintain a certain grain size and crystal phase (Zheng Y. F. et al., 2020). Yu *et al.* prepared the defective N-doped Co_3_O_4_ by efficient N_2_ plasma treatment for methane oxidation reaction. N-doped Co_3_O_4_ could synergistically boost the catalytic performance by increasing active surface oxygen, enhancing redox property, and promoting the C–H bond activation ability. This result may provide a valuable guidance for the defects engineering of Co_3_O_4_ for the application of CMC (Yu et al., 2020).

However, doping is not always favorable for the catalytic activity of Co_3_O_4_. Choya *et al.* prepared two bulk Co_3_O_4_ catalysts with and without residual sodium by precipitation method. It was found that the presence of Na^+^ had a negative impact on the properties of the Co_3_O_4_ catalyst due to diffusion and migration into the spinel lattice. The insertion of Na^+^ led to lattice distortion and induced a reduction of the Co^3+^ into Co^2+^ owing to high Lewis acidic properties, along with high electron density within the oxygen ions of the lattice, weakened Co–O bonds, and reduced lattice oxygen species, which pointed out that the doping may be detrimental to catalytic performance. In this sense, appropriate precipitants or preparation methods are important for achieving high-performance CMC catalysts (Choya et al., 2018b).

#### 3.1.3 Binary Co_3_O_4_-catalysts

Li *et al.* reported the introduction of an electric field on the Co_3_O_4_/Ce_0.75_Zr_0.25_ catalyst and found that the presence of the electric field significantly promoted the catalytic oxidation activity of methane. Co_3_O_4_ provided active sites and oxygen species for methane oxidation, while CeO_2_ released lattice O species for the oxidation of CoO and Co to Co_3_O_4_. The electric field promoted the reduction of Ce^4+^ to Ce^3+^ and promoted the release of oxygen from the lattice. The chemisorption of methane mainly located in the newly formed tetrahedral Co^3+^ in the electric field rather than the active sites formed by octahedral Co^3+^ with gaseous oxygen. The adsorbed CH_4_ was oxidized to carbonates species immediately, followed by the formation of CO_2_ and refreshment of consumed O species by gaseous oxygen, which is similar to the typical MvK mechanism (Li L. et al., 2019).

#### 3.1.4 Deactivation

Although Co_3_O_4_ shows a high catalytic activity for the CMC, it still cannot defeat noble metals owing to its low water and sulfur resistance. Generally, the inhibition of water vapor on the catalytic activity is probably due to its competition with the reactant for the active sites. The inhibition of SO_2_ is attributed to the formation of sulfate or sulfide on the surface of catalysts, which may cause the loss of active sites, or agglomeration and the loss of surface area. From the existing literature, this problem can be effectively solved when the TMOs are supported on the carriers or some additives are added to the TMOs (Li J. A. et al., 2019).

Li *et al.* prepared a series of CoO_x_ embedded in the porous SiO_2_ matrix by a spontaneous deposition method, which showed enhanced catalytic activity as compared with the simply supported CoO_x_/SiO_2_ catalyst due to a higher ratio of O_ads_/O_latt_ and more active sites obtained by embedded CoO_x_/SiO_2_. Due to the competitive adsorption of water and reaction molecules on the active sites, water vapor inhibits the catalytic efficiency. While when H_2_O was removed, the CH_4_ conversion nearly fully recovered. Under moisture conditions, the embedded CoO_x_@SiO_2_ exhibited a high thermal stability and efficient moisture resistance due to the silica encapsulation (Li K. et al., 2019).

#### 3.1.5 Mechanisms

According to the available mechanism study on CMC over TMOs, CMC over Co_3_O_4_ usually follows the MvK mechanism, which means that CH_4_ is actually oxidized by the oxygen species present in the Co_3_O_4_ lattice, followed by the generation of oxygen vacancies. Then, the O_2_ from the gas phase refills the oxygen vacancies, provoking the subsequent reoxidation of the Co_3_O_4_-basedcatalyst. Wu *et al.* prepared Co_3_O_4_–CeO_2_ mixed oxides using (NH_4_)_2_CO_3_, Na_2_CO_3_, and CO(NH_2_)_2_ as precipitation agents by a precipitation method. The Co_3_O_4_–CeO_2_ with the homogeneous precipitation of CO(NH_2_)_2_ showed excellent catalytic activity owing to the small crystallite size, easy reducibility of Co^3+^, and high surface Co^3+^ content at the Co_3_O_4_–CeO_2_ interface. The peculiar structure and morphology of CeO_2_ played a fundamental role in stabilizing the Co_3_O_4_ active phase against sintering and promoting its activity. The authors believed that the pathway of CMC over the Co_3_O_4_–CeO_2_ was in accordance with the MvK mechanism, in which the Ce^4+^/Ce^3+^ couple efficiently released oxygen (Wu et al., 2015).

**FIGURE 4:**
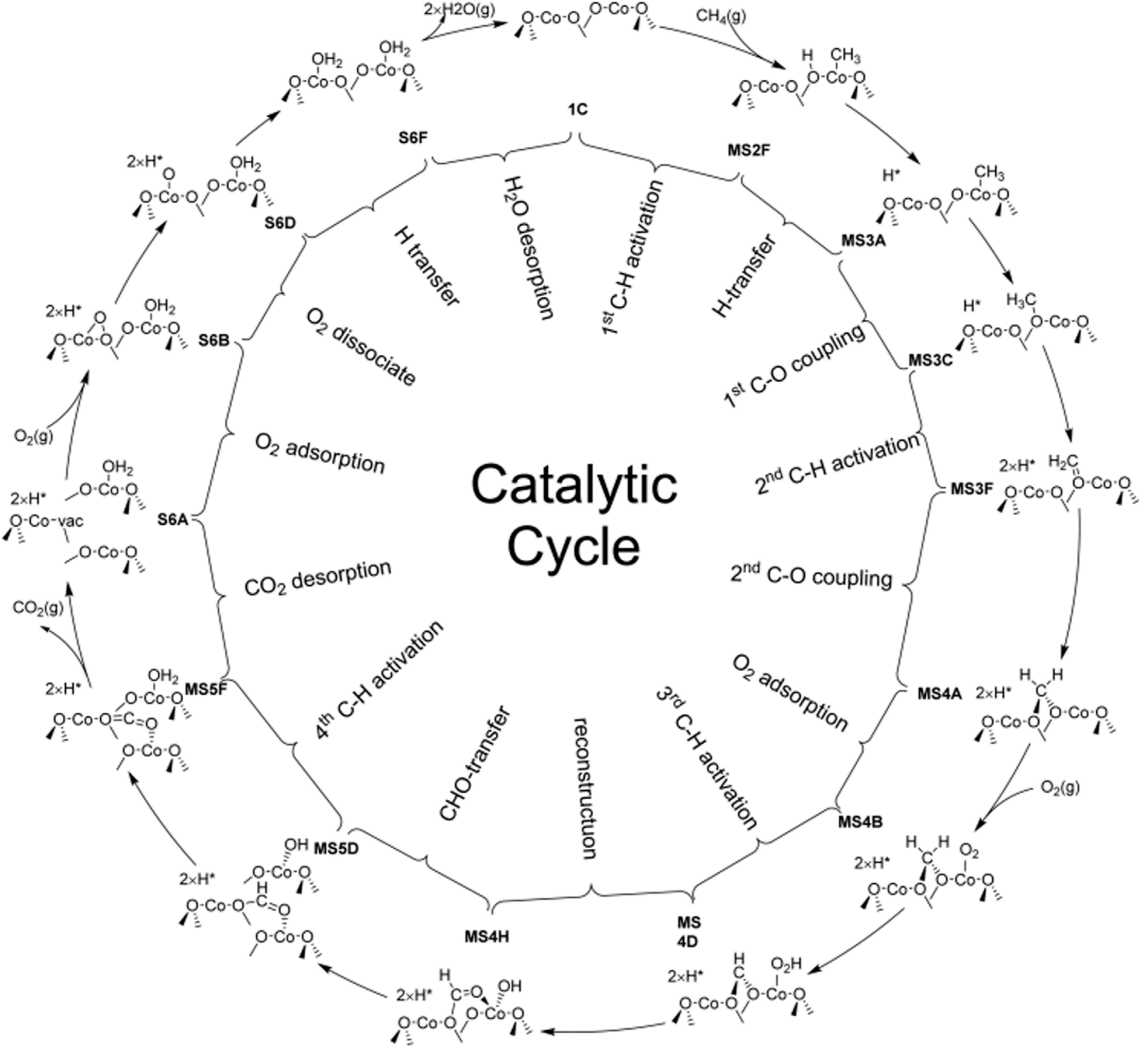
Schematic diagram of the whole catalytic cycle of CMC over Co_3_O_4_ (110) following the optimal reaction mechanism. Reprinted with permission from Hu et al. (2016). Copyright (2016) American Chemical Society.

Hu *et al.* investigated CMC on the Co_3_O_4_(110) surface with excellent catalytic performance by first-principles calculations as compared with that on the Co_3_O_4_(100) surface. It is found that the optimal reaction pathway of CMC over Co_3_O_4_(110) would be CH_4_ → CH_3_* → CH_3_O_2c_→ CH_2_O_2c_ → O_2c_CH_2_O_2c_ → O_2c_CHO_2c_ → O_2c_CHO* →CO_2_, in which the 2-fold coordinated lattice oxygen (O_2c_) was the key to the first two C–H bond activations and the C–O bond coupling (Figure 4). According to the figure, H is able to readily transfer swiftly among different surface oxygen species to form adsorbed H_2_O* for the rapid regeneration of active O_2c_. The cooperation of multiple active sites not only facilitates the H swift transfer in order to maximally prevent the passivation of the active low-coordinated O_2c_ but also stabilizes surface intermediates during the CMC. Due to the synergistic effect of surface-adsorbed oxygen and lattice oxygen, the reaction pathway belongs to the T-T mechanism, in which the first C–H bond activation step would be the rate-determining step for the CMC on the Co_3_O_4_ (110) surface (Hu et al., 2016).

However, some researchers have proposed different mechanisms for the CMC over Co_3_O_4_. Han *et al.* prepared a series of Co_3_O_4_ nanocrystals with different morphologies *via* calcining the Co(OH)_2_ precursor at different temperatures, among which the mesoporous Co_3_O_4_ possessed excellent catalytic activity due to the vacancy, antisite, dislocation, and grain boundary defects in the narrow junction regions and the structure of the pore wall. The authors believed that the reaction pathway followed the E–R mechanism, however, without detailed verification (Han et al., 2016).

### 3.2 NiO-based catalysts

#### 3.2.1 Single NiO

NiO, NaCl-type cubic spar structure, is a typical P-type semiconductor with the intrinsic defects at the Ni^2+^ metal sites, which give rise to positive holes (p^+^) to generate Ni^3+^ (Ni^2+^ + p^+^→Ni^3+^) or O^−^ species (O^2−^ + p^+^→O^−^). NiO is extensively used in the field of catalysis, chemical sensors, battery electrodes, and magnetic and electronic devices due to excellent properties such as catalytic activity, thermo-sensitivity, and super-paramagnetic property (Wang et al., 2021). Especially, in the field of catalysis, NiO-based catalysts attract a lot of attention due to the surface electrophilic O^2−^ species being effective for the activation of C–H bonds. NiO have been successfully prepared *via* a variety of methods, including chemical precipitation, electrode deposition, sol–gel technique, surfactant-template, hydrothermal technique, template-free strategy, microwave-assisted gas/liquid interfacial method, and solvothermal method.

Ye *et al.* prepared polymorphous NiO with different morphologies, including nanoparticle-based sheets, octahedra, nanosheet-built agglomerates, and nanoparticle-based microsphere, *via* a simple one-pot thermal decomposition approach. The morphology and crystal properties of NiO can conveniently be achieved by selecting various decomposition temperatures and precursors. The nanoparticle-based sheets and nanosheet-built agglomerates showed a high catalytic performance owing to the small crystal size and large specific surface area by using NiC_2_O_4_·2H_2_O and NiCO_3_·2Ni(OH)_2_·4H_2_O as precursors (Ye et al., 2016).

Chen *et al.* prepared NiO-NSL nanomaterials with a characteristic nanorod structure through the solid–liquid NH_3_·H_2_O precipitation method. The content of Ni^2+^ on the surface of NiO-NSL was higher than traditional NiO-based catalysts, consistent with DFT calculations in which the energy barrier for the C–H bond activation on Ni^2+^ was lower than that on Ni^3+^. However, the authors did not clearly explain the mechanism of the CMC on the NiO-NSL catalyst, but only highlighted the significant impact of Ni^2+^ on the catalytic performance of the NiO-based catalysts (Chen K. et al., 2021).

#### 3.2.2 Doped NiO

Generally, the synergistic effect induced by the doping can significantly improve catalytic activity. Zhang *et al.* prepared a series of MnO_x_–NiO composite oxide catalysts by the co-precipitation method, which exhibits higher catalytic performance compared with the single NiO and MnO_x_. The characterization results demonstrate that the Ni–Mn–O solid solution formed by the doping of appropriate amount of Mn, showing abundant highly dispersed Mn^4+^ and higher coordination number as well as certain nickel vacancies, due to the synergy interaction of Ni and Mn (Zhang et al., 2013).

#### 3.2.3 Binary NiO-catalysts

**FIGURE 5:**
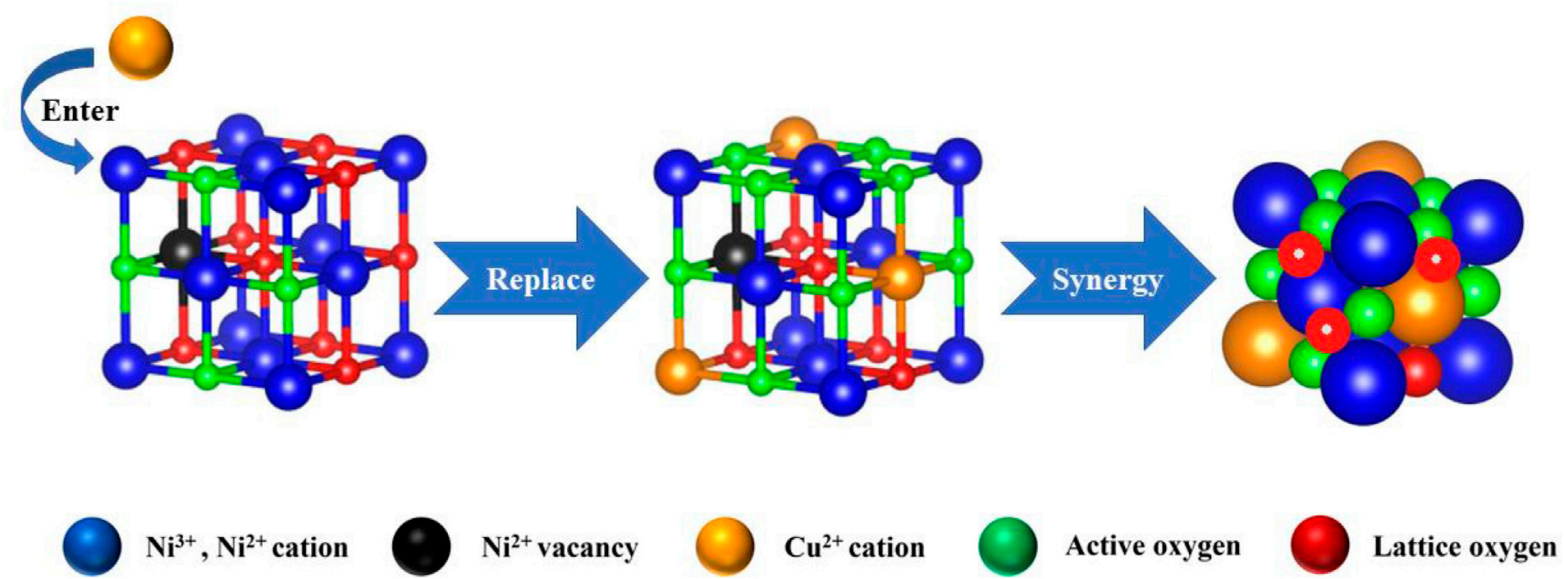
Reaction pathway of CMC over Cu–Ni oxide. Cited from Fan et al. (2022).

Fan *et al.* introduced Cu into the NiO lattice to generate the Cu–Ni solid solution with the mesoporous structure by the co-precipitation method. Ni–Cu oxide catalysts manifested superior catalytic activity, moisture tolerance, and durability, owing to more unsaturated Ni atoms and lattice defects. Ni–O–Cu bonds could weaken Ni–O and Cu–O bonds, making the lattice oxygen converted into adsorbed oxygen efficiently and creating more oxygen vacancies to promote the activation and adsorption capacity of Ni–Cu oxide for O_2_, creating more surface electrophilic species (O_2_^2−^, O_2_^−^ and O^−^). Thus, the reaction pathway was in agreement with the MvK mechanism (Figure 5). The electron densities were redistributed because of the interaction between NiO and CuO, where the surface acid–base properties were adjusted. The higher basicity over Cu–Ni oxide could contribute to the adsorption of CH_4_ with weak acidity and inhibit the accumulation of surface hydroxyl groups effectively. Meanwhile, the stronger surface acid sites could facilitate the activation of CH_4_ *via* heterolytic C–H bond breaking (Fan et al., 2022).

#### 3.2.4 Deactivation

Through the regulation of preparation method and structure, Ni-based catalysts can obtain excellent resistance to water vapor. Liu *et al.* prepared the simple metal oxides (Fe_2_O_3_, Co_3_O_4_, NiO, and CuO) by the thermal decomposition of the corresponding metal nitrates. The NiO possessed larger capability for oxygen adsorption, thus exhibiting the best catalytic activity for CMC as compared to other metal oxides, even superior to the perovskite catalyst LaCoO_3_. Water vapor promoted CH_4_ conversion over the NiO catalyst, which could be attributed to H_2_O could modify NiO surface and promote the activation of O_2_ and CH_4_ on the surface (Liu et al., 2017). Xu *et al.* synthesized NiO catalysts with the mesoporous structure by hydrothermal method with polyethylene glycol (NiO-PEG) and polyvinyl pyrrolidone (NiO-PVP) as soft templates. NiO-PEG and NiO-PVP possessed more mobile-active oxygen species, which was beneficial to the activating of the CH_4_, due to the mesoporous structure and high surface area. NiO-PEG displayed excellent reaction stability in the presence or absence of water vapor for the reason that its bulk structure possessed certain physical stability (Xu et al., 2017).

#### 3.2.5 Mechanisms

**FIGURE 6:**
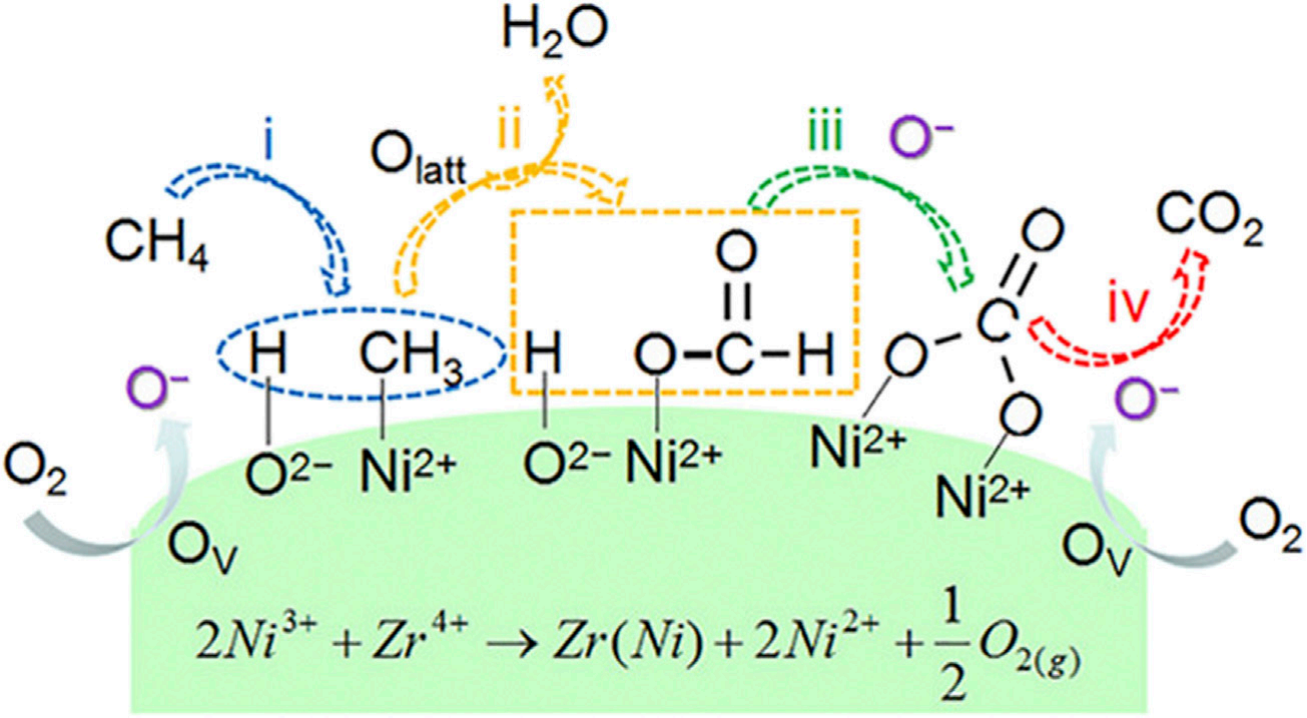
Reaction pathway for methane combustion over Ni_1−x_Zr_x_O_2−δ_ catalysts. Reprinted with permission from Wang et al. (2021). Copyright (2021) American Chemical Society.

It is generally believed that the pathway of the CMC over nickel-based catalysts is consistent with the MvK mechanism. Shu *et al.* prepared mesoporous NiO with abundant oxygen defects by a NaCl crystalline scaffold-based method. In kinetic measurements, r_CH4_ exhibited a near zero-order dependence on O_2_ partial pressure, indicating that CH_4_ oxidation was not sensitive to the P_O2_ partial pressure. Due to the depletion of lattice oxygen, the concentrations of C^16^O^16^O and C^18^O^16^O were reduced, and the concentration of C^18^O^18^O was increased, which further demonstrated that the reaction mechanism was accorded with the MvK mechanism. In this case, the highly abundant lattice oxygen species had great contribution to the excellent performance of NiO (Shu et al., 2022). Wang *et al.* prepared Zr-promoted NiO nanocatalysts by a designed co-precipitation process. Ni_0.89_Zr_0.11_O_2-δ_ solid solution phase exhibited abundant active Ni^2+^ sites and oxygen vacancies, bringing about the increase in surface acidic–basic sites. The mechanism for CMC could be described, as shown in Figure 6. CH_4_ was adsorbed on the Ni^2+^–O^2−^ active site at first and then dissociated to -CH_3_ and -OH, followed by the formation of formate and carbonate intermediates. Then, the carbonate species converted to CO_2_. The authors did not mention which mechanism the CMC on Ni_0.89_Zr_0.11_O_2-δ_ conforms to, but according to the lattice oxygen and oxygen vacancies involved in the reaction, it is judged that the reaction conforms to the MvK mechanism. Meanwhile, in the stream tests, the steady conversion of CH_4_ could be well maintained regardless of the presence of H_2_O due on the doping of Zr (Wang et al., 2021).

### 3.3 MnO_2_-based catalysts

#### 3.3.1 Single MnO_2_

Mn oxides, such as α- and β-MnO_x_, possess the different polymorphic structures, which exhibit mixed valence state of Mn (Mn^2+^, Mn^3+^, and Mn^4+^), and different ways to link together the basic octahedral [MnO_6_] units, thereby showing strikingly different and efficient catalytic activities for the CMC (Jia J. B. et al., 2016; Yang et al., 2021). The catalytic activity of the Mn oxides is typically determined by their prepare method, morphology, crystal structure, and degree of oxidation.

Yu *et al.* prepared γ-MnO_2_ with abundant surface and lattice defects by a combined ball-milling and selective atom removal method, which showed a high catalytic activity for the CMC owing to the enhanced specific surface area, high Mn^4+^/Mn^3+^ ratio, more active oxygen species, and enhanced reducibility (Yu et al., 2019). Wasalathanthri *et al.* prepared mesoporous amorphous Meso-Mn-A, Meso-Mn_2_O_3_, Meso-ε-MnO_2_ (epsilon phase), and octahedral molecular sieves MnO_2_ (Meso-OMS-2) *via* an inverse surfactant micelle method. Meso-OMS-2 showed the highest catalytic activity, attributing to the narrow and monomodal pore size distribution, higher surface area, the oxidation states, and surface oxygen vacancies, promoting the lattice oxygen mobility, following the MvK mechanism (Wasalathanthri et al., 2015).

#### 3.3.2Doped MnO_2_

In addition, compared to pure MnO_x_, the doping of other transition metals will also significantly enhance the catalytic performance of Mn-based oxides. Neatu *et al.* studied CeO_2_–MnO_x_ catalysts by three methods, illustrating that the CeO_2_–MnO_x_ catalyst impregnated on the support surface showed superior catalytic activity as compared with the catalyst doped into the bulk. The authors believed that the CMC over the CeO_2_–MnO_x_ catalyst was in accordance with the MvK mechanism, in which the oxidation of CH_4_ took place using the lattice oxygen, consecutively with the reduction of Ce^4+^ and Mn^4+^ to Ce^3+^ and Mn^3+^, respectively. The gaseous O_2_ was used to reoxidize the surface to assure another catalytic cycle (Neatu et al., 2019).

#### 3.3.3 Deactivation

**FIGURE 7:**
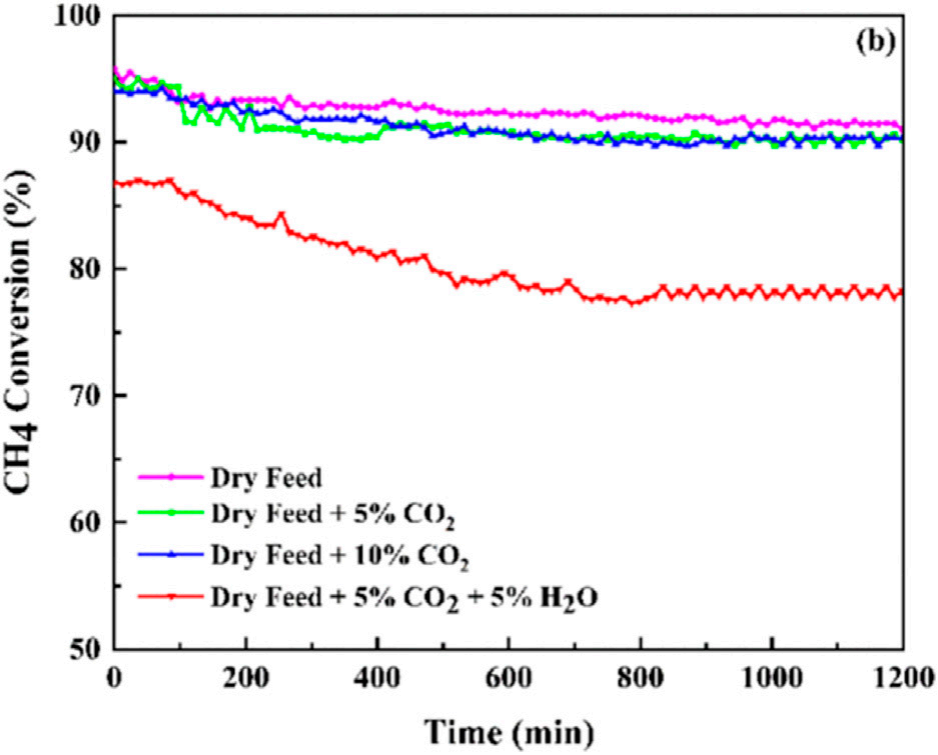
Catalytic stability for methane catalytic combustion of the MnO_2_ catalyst under different feed compositions. Reprinted with permission from Akbari et al. (2021). Copyright (2021) American Chemical Society.

However, water vapor will affect the catalytic activity of Mn-based catalysts for the CMC to some extent. Akbari *et al.* prepared nanostructured MnO_2_ catalysts with various morphologies by the simple hydrothermal method and the solution method, among which the α-MnO_2_ catalyst with a wire-like morphology exhibited the best performance for CMC. However, the stability of the optimal MnO_2_ sample reduced to some extent with the water vapor in the reactant feed stream (Figure 7). On one hand, the coverage of the active sites by water vapor inhibited the adsorption of CH_4_ and O_2_ on the MnO_2_ catalyst surface. On the other hand, the -OH group as an inert compound over lattice oxygen could also cause a decline in the catalytic stability (Akbari et al., 2021).

**FIGURE 8:**
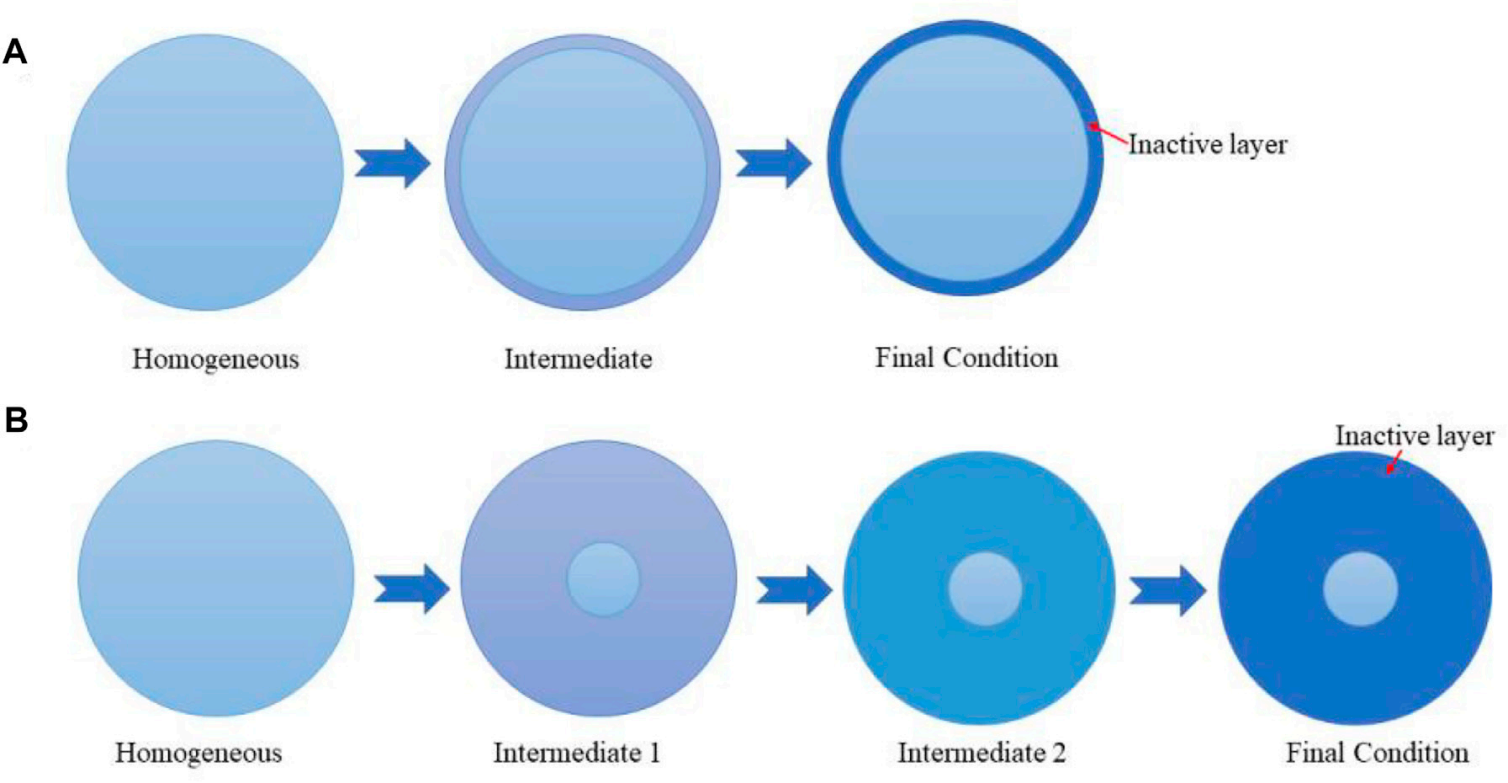
SO_2_-poisoning mechanism over MnCe-CP **(A)** and MnCe-RP **(B)**. Cited from Zhong et al. (2019).

If in an oxidizing atmosphere, SO_2_ will be oxidized to form sulfate, which will be deposited on the surface of MnO_2_, hindering the multiple adsorption, activation, and oxidation of CH_4_ over MnO_2_, then causing the deterioration of catalytic activity. Zhong *et al.* synthesized MnCe-RP and MnCe-CP by the redox-precipitation (RP) and co-precipitation (CP) methods, respectively, and studied SO_2_ resistance of the catalysts (Figure 8). The SO_2_ would decrease the content of lattice oxygen and Mn^4+^, so the CH_4_ conversion of MnCe-CP reduced by 62.45%. On the contrary, the CH_4_ conversion of MnCe-RP only reduced by 1.08% owing to the excellent morphology and the redox potential of K_x_Mn_8_O_16_, absorbing and oxidizing SO_2_ to sulfides, freeing from the poison of the downstream catalyst (Zhong et al., 2019).

#### 3.3.4 Mechanisms

There are reported contradictory claims on the CMC over Mn oxides, such as that based on the L–H mechanism involving adsorbed oxygen and that based on the MvK mechanism concerning lattice oxygen.

Zhang *et al.* synthesized nanocubic MnO_2_ (MnO_2_-C) with planes (101) and nanorod-shaped MnO_2_ (MnO_2_-R) with planes (110) by a hydrothermal process, illustrating that the morphologies of MnO_2_ had significant influence on the catalytic performances of CMC. Due to higher BET surface area, smaller crystalline size, more surface oxygen vacancies, and better low-temperature reducibility, MnO_2_-C displayed higher catalytic performances and good thermal stability compared with MnO_2_-R. The authors believed that the surface oxygen of MnO_2_-C could react with the adsorbed C–H to generate the carboxylate species for the reason of more surface oxygen vacancies, followed by the oxidation of carboxylate species to CO_2_, through the L–H mechanism (Zhang et al., 2019). Jia *et al.* synthesized two types of single-phase manganese oxides MnO_2_ with different levels of nonstoichiometric defects, α-MnO_2_ (Mn1) and γ-MnO_2_ (Mn2), compared with a stoichiometric Mn_2_O_3_ (Mn3) as a reference. Results revealed that both Mn3 and Mn1 exhibited enhanced activities due to more structural defects. It was found that CH_4_ was directly oxidized to carbonates on the surface of the pre-oxidized Mn1 and subsequently transformed to CO_2_ in the absence of gaseous O_2_ by the *in situ* DRIFTS and CH_4_-TPD test, which showed that the active oxygen involved in the CH_4_ oxidation was largely derived from the surface lattice oxygen of the Mn1, in accordance with the MvK mechanism. The O_2_ on–off profiles further demonstrate reaction mechanism, in which the desorbed CO_2_ was generated by the reaction between CH_4_ and lattice oxygen from Mn1 without O_2_, while the increase in CO_2_ resulted from the reaction between CH_4_ and the constantly refilled oxygen at the vacancies in the presence of O_2_ (Jia et al., 2019).

Wang *et al.* studied the roles of the crystallographic structure of Mn-based oxides, among which α-MnO_2_ exhibited the superior catalytic performances, attributing to higher surface Mn concentration and more active oxygen species, more mono-μ-oxo bridged (corner-shared) MnO_6_ sites, and better reducibility. CMC over the MnO_2_ catalyst proceeds *via* both MvK and L–H mechanisms, while the latter was predominant, in which the dehydrogenation of CH* to H* was considered as the rate-determining step and intermediate species -COO and -CH_3_O were oxidized by active oxygen species to CO_2_ and H_2_O (Wang et al., 2018).

### 3.4 Cr_2_O_3_-based catalysts

#### 3.4.1 Single Cr_2_O_3_

The Cr_2_O_3_, trigonal crystal system belongs to the α-Al_2_O_3_ structure, which is composed of oxygen ions as the closest hexagonal packing and Cr^3+^ filling the octahedral gap formed by these close packings. The coordination number of Cr^3+^ is 6, and only 2/3 of the octahedral gap is occupied by chromium ions. The activity and selectivity of Cr_2_O_3_ are closely related to the change of the valence state of chromium ions. Various techniques have been developed to prepare Cr_2_O_3_ nanoparticles such as co-precipitation, incipient wetness impregnation, sol–gel, solvothermal, solid thermal decomposition, and sonication (El-Sheikh et al., 2009).

Jodłowski *et al.* applied the sonication method to prepare transitional metal oxide catalysts (Co, Cu, Cr), among which the chromium oxide catalyst showed greater catalytic activity as compared to cobalt oxide, copper oxide, and commercial palladium catalysts, attributing to the presence of a high concentration of formate on the surface of chromium oxide (Jodłowski et al., 2016).

#### 3.4.2 Doped Cr_2_O_3_

The doping of Cr_2_O_3_ enables the catalyst to exhibit excellent catalytic activity due to the synergistic effect between TMOs. Yuan *et al.* prepared Cr-based catalysts modified by Ce *via* incipient wetness impregnation method and investigated the effects of the Ce loading amount. It was found that the Cr-based catalysts with 3 wt% Ce showed higher catalytic performance due to an increasing amount of reaction site [CrO_4_] species, improving the high-temperature-resistant performance and obtaining the synergistic effect between Ce and Cr (Yuan et al., 2013). Dupont *et al.* synthesized Cu/Cr oxides with high specific surface area by the sol–gel route using propionic acid. The results suggested that Cu/Cr oxides prepared by the special sol–gel process showed better catalytic performances compared with Cu/Cr oxide commercial catalysts, attributing to well-dispersed Cu species and the surface enrichment with Cr^6+^. In addition, though DFT coupled with periodic slab models, the authors believed that O_2_ molecules dissociatively adsorbed over the Cu/Cr oxide surface at the first step, along with generating the active oxygen species mainly at the Cr sites (Dupont et al., 2010).

#### 3.4.3 Deactivation

Due to its poor affinity for acid gases, Cr_2_O_3_ exhibits excellent resistance to sulfur poisoning. Ordo´n˜ez *et al.* prepared different bulk metal oxides (Cr_2_O_3_, Co_3_O_4_, Mn_2_O_3_, NiO, and CuO) and investigated their catalytic stability for CMC in the presence of SO_2_. It was found that Cr_2_O_3_ exhibited highly sulfur-tolerant, whereas the other materials were deactivated rapidly, even if Co_3_O_4_ and Mn_2_O_3_ were more active than Cr_2_O_3_ for CMC in the absence of sulfur species. Characterization results illustrated that the excellent stability was caused by the low affinity of Cr_2_O_3_ to acid gases (as SO_2_); thus, sulfates would not form on the surface of the catalyst to occupy the active sites (Ordóñez et al., 2008).

#### 3.4.4 Mechanisms

There is still controversy about the mechanism of CMC on Cr_2_O_3_. Huang *et al.* synthesized a homogeneous ZnCr_2_O_4_ oxides by the ethylene glycol-mediated solvothermal method. Cr^3+^ and Cr^6+^ coexisted in ZnCr_2_O_4_, in which Cr^6+^ probably caused the presence of interstitial oxygen species in the structure. The authors believed that the reaction pathway of CMC over the ZnCr_2_O_4_ catalyst was consistent with the L–H mechanism under low temperature, in which the interstitial oxygen was involved to the methane combustion. However, the mechanism has not been experimentally verified (Huang et al., 2019).

On the contrary, Zhu *et al.* applied the co-current co-precipitation method to prepare a series of Sn–Cr binary oxide catalysts among which the oxide with a Cr/Sn atomic ratio of 3 : 7 showed excellent catalytic activity due to higher surface areas and high oxidation states of chromium ions. Temperature-programmed ^18^O isotope-exchange measurements confirmed that CMC over Sn–Cr binary oxide catalysts occurred *via* a redox cycle with the chromium ion as the active center, following the MvK mechanism (Zhu et al., 2003).

### 3.5 Fe_2_O_3_-based catalysts

#### 3.5.1 Single Fe_2_O_3_

Fe_2_O_3_ is an earth-abundant and non-toxic material that has been extensively used to catalyze a wide range of reactions including water gas shift reaction, photocatalytic water splitting, looping combustion, etc. There are four polymorphs of Fe_2_O_3_, which are Hematite (α-Fe_2_O_3_), Maghemite (γ-Fe_2_O_3_), β-Fe_2_O_3_, and ε-Fe_2_O_3_. Among which α-Fe_2_O_3_ is thermodynamically stable and has the corundum(α-Al_2_O_3_) structure, which is based on a hcp anion packing. γ-Fe_2_O_3_ is an inverse spinel structure with cation-deficient sites and can be transformed from α-Fe_2_O_3_ at high temperature. β-Fe_2_O_3_ andε-Fe_2_O_3_ have been synthesized only in the laboratory. The former has been obtained by the de-hydroxylation of β-FOOH under high vacuum at 170°C. The structure of ε-Fe_2_O_3_ is intermediate between those of α-Fe_2_O_3_ and γ-Fe_2_O_3_. It can be prepared in various ways and transforms to α-Fe_2_O_3_ at between 500 and 750°C, apparently according to the method of preparation (Cornell and Schwertmann, 2003).

Among the few researches dealing with the use of Fe_2_O_3_ in CMC, α-Fe_2_O_3_ was the dominant catalytic active phase, and preparation method affects its catalytic performance greatly. Barbosa *et al.* synthesized bulk α-Fe_2_O_3_ by precipitation (α-Fe_2_O_3_-p) and the citrate method (α-Fe_2_O_3_-c), finding that the preparation method strongly influences both the initial activity of the catalyst and its stability under reaction conditions (Barbosa et al., 2001). The α-Fe_2_O_3_-p catalyst presented higher surface areas, in correlation with greater initial activity and lower light-off temperatures than that of the α-Fe_2_O_3_-c catalyst. Although all catalysts undergo sintering at the high operation temperature with the loss of active sites, the α-Fe_2_O_3_-p catalyst exhibited less sintering and better stability. On this basis, Paredes *et al.* prepared α-Fe_2_O_3_ base catalysts *via* the acid dissolution-alkaline precipitation method using red mud as a raw material, an aluminum industrial waste formed by α-Fe_2_O_3_, Ti, Al, Ca, and Na, and compared the CMC performance and reaction mechanism with unprocessed red mud, and massive α-Fe_2_O_3_ synthesized by the precipitation method (α-Fe_2_O_3_-c mentioned above) (Paredes et al., 2004). For the combustion of 2000 ppm V CH_4_ in air, the α-Fe_2_O_3_-c exhibits the best activity (T_50_ = 461°C), and the activity of processed red mud (T_50_ = 530°C) is much higher than that of unprocessed red mud (T_50_>650°C) because after treatment the component of Na and Ca, which can hinder the catalyst activity decreases. Moreover, when conversion per Fe content in the catalyst is considered, the difference between α-Fe_2_O_3_-c and processed red mud catalysts is much smaller at low conversions. This would indicate that the other constituents of red mud have little or no effect on the catalyst activity. As for the mechanism, the CH_4_-TPD experiments (100–600°C) suggested that very little CH_4_ is adsorbed, the reticular oxygen of the catalyst being enough for its complete oxidation.

In addition, researchers have achieved the catalytic conversion of CH_4_ over α-Fe_2_O_3_ catalysts with different morphologies. Dong *et al.* fabricated 3D urchin-like mesoporous α-Fe_2_O_3_ nanoarchitectures with the combination of nonhomogeneous ionic liquid/diphenyl ether solvothermal method and solid-state thermal annealing. They found that CH_4_ was converted into products containing C–O bonds (CO_2_) at 230°C, which is 190°C lower than over bulk α-Fe_2_O_3_. As for the reason, with the measurement and comparison of adsorbed oxygen on the two material, they inferred that the urchin-like α-Fe_2_O_3_ nanoarchitectures have a higher density of surface oxygen vacancies than bulk α-Fe_2_O_3_, which can accelerate the dissociation of oxygen molecules at the surface and increase the mobility of lattice oxygen (Dong et al., 2014). It should be noted that this structure does not exhibit good CH_4_ conversion at high temperatures (700°C, conversion = 16%). He *et al.* invented a hard-templating synthetic strategy to guide the anisotropic growth of ultrathin α-Fe_2_O_3_ nanosheets with a large (110) facet exposure ratio, the catalytic performance of which in low-temperature CH_4_ combustion is comparable to that of noble metal-based catalysts (He L. et al., 2020). The antiferromagnetic coupled diiron core on the (110) crystallographic plane of α-Fe_2_O_3_ is a structurally favorable condition, which resembles the diiron active site in soluble CH_4_ monooxygenase, an enzyme that converts CH_4_ to methanol in nature (Ross and Rosenzweig, 2017). Meanwhile, they utilized oxygen isotopic tracing experiments and DFT calculations to confirm that CH_4_ is primarily activated by lattice oxygen below 400°C, in accordance with the MvK mechanism. They further speculated that at higher temperatures, CH_4_ would be activated predominantly by molecular oxygen instead, as the accumulation of lattice oxygen vacancy favoring the adsorption of molecular oxygen from the gas phase (Cheng et al., 2016).

#### 3.5.2 Deactivation

**FIGURE 9:**
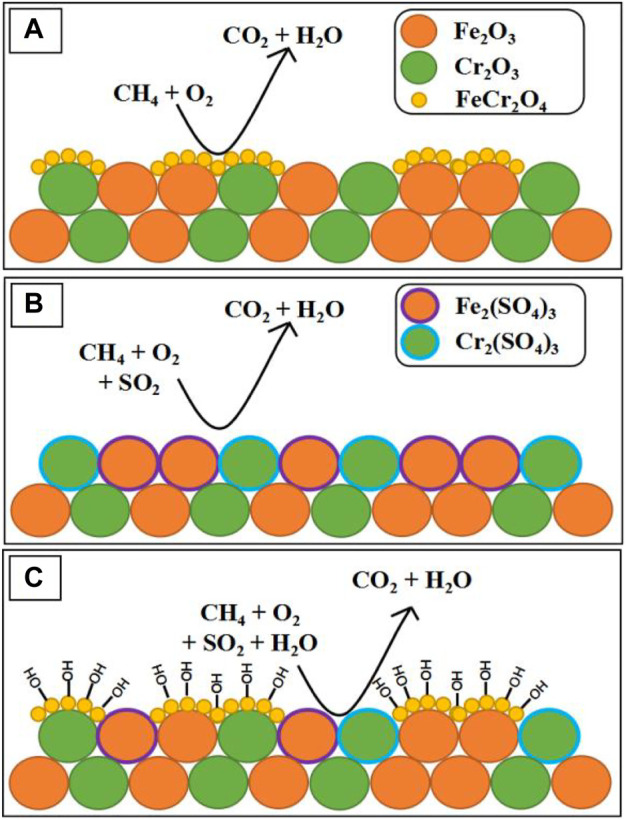
Proposed poisoning mechanisms at 450°C under **(A)** sulfur-free conditions, **(B)** dry conditions, and **(C)** wet conditions. Cited from García-Vázquez et al. (2020).

CH_4_ emissions are often accompanied by large quantities of steam and traces of sulfur-containing gases, which are the two most performance hindering species present in typical CH_4_ after-treatment operating conditions (Raj, 2016). Setiawan *et al.* confirm that α-Fe_2_O_3_ catalysts are significantly lower activity under the mixture conditions of water vapor and CH_4_ due to the strongly bound between water (hydroxyl) species and Fe_2_O_3_, which destruct the active sites irreversibly (Setiawan et al., 2015). Recently, García-Vázquez *et al.* synthesized iron- and chromium-based oxides by using the citrate sol–gel method and investigated their catalyst performance of CMC in the presence of SO_2_ and steam. They found that catalyst Fe_60_Cr_40_ (molar ration) exhibited the remarkable performance (366 ppm CH_4_ balanced in air, T = 450°C, conversion = 79%) due to the formation of the FeCr_2_O_4_ spinel phase, which is the most active sites in these catalyst for CMC. And because the FeCr_2_O_4_ spinel phase can still exits after being aged under wet conditions, the Fe_60_Cr_40_ showed the better catalyst conversion of CH_4_ than α-Fe_2_O_3_ catalyst, which can also be found by comparing Figures 9A,B (312 ppm CH_4_ + 4.6 ppm SO_2_ 10% vol H_2_O balanced in air, T = 450°C, conversion = 47% vs. 26%). In addition, as has been represented in Figure 9C, the competitive adsorption of hydroxide groups on FeCr_2_O_4_ even slows down the rate at which sulfur dioxide poisons the catalyst’s active sites (García-Vázquez et al., 2020).

#### 3.5.3 Mechanisms

In conclusion, the reaction of α-Fe_2_O_3_ catalyst in CMC involves the MvK mechanism. Recently, Tang *et al.* adopted the generalized gradient approximation (GGA) + U approach to investigate the reaction pathways of complete and partial oxidations of CH_4_ on the dominant Fe–O_3_–Fe termination of thermodynamically stable hematite (α-Fe_2_O_3_) (0001) facets. The energy barrier for the first C–H bond activation is 1.04 eV. In the transition state, the dissociating H is in contact with the lattice O and with the dissociating CH_3_ is in contact with the Fe site. Subsequent decomposition and oxidation of the CH_x_ species (x = 1, 2, 3) exploit the lattice O species according to the MvK mechanism, forming CH_x_O in more thermodynamically and kinetically favorable pathways. For the two pathways, the overall rate-limiting steps are both the first C–H bond activation. Adsorption of O_2_ on the VO site is exothermic (-1.42 eV), with one O atom binding at the VO site and the other O binding on the Fe site. In particular, after the dissociation of O_2_ *via* the O–O bond cleavage, one O fills the oxygen vacancy, and the other O became the ferryl O (Fe=O). Although the ferryl O is highly active and capable of lowering the energy barrier of the C–H bond activation, the availability of extremely active ferryl O is expected to be too low on the Fe–O_3_–Fe-terminated surface to critically impact the overall catalyst performance (Tang and Liu, 2016).

### 3.6CeO_2_-based catalysts

#### 3.6.1 Single CeO_2_

CeO_2_ is the most stable form of cerium oxide, which has a face-centered cubic structure and space group of Fm-3m (Trovarell, 2002). The coordination of cerium is 8 and that of oxygen is 4, which means that there are large vacant octahedral holes in the structure. Because of its structure, CeO_2_ has an excellent storage-release capacity in a reversible manner. Specifically, Ce^4+^ can be reduced to Ce^3+^ under anoxic conditions, accompanied by a rapid release of lattice oxygen from the solid to the gas phase, leaving oxygen vacancy defects. On the other hand, Ce^3+^ can be re-oxidized into Ce^4+^ by adsorbing oxygen under oxygen-rich conditions and therefore refills the vacancies. Such unique redox properties render CeO_2_ a wonderful support for noble metal catalysts in the catalytic oxidation of hydrocarbons, following mostly the MvK mechanism (Aneggi et al., 2016).

#### 3.6.2 Doped CeO_2_

CeO_2_ has been actively studied in the past few decades as an alternative to noble metal catalysts for CMC (Mukherjee et al., 2016). While pure CeO_2_ phase exhibits a poor catalytic activity at low temperatures and a poor thermal stability at high temperatures, which significantly hampers its industrial uses (Kašpar et al., 1999). Efforts have been made to improve the catalytic CH_4_ combustion performance of CeO_2_-based catalysts such as compositing with other metal oxides, heteroatom doping, etc. For instance, more oxygen vacancies can be introduced into the CeO_2_ structure by doping cations with oxidation states below or equivalent to 4^+^ such as Ca^2+^, Mn^2+,^ Ni^2+^, Cu^2+^, Fe^3+^, Co^3+^, La^3+^, and Y^3+^ (Palmqvist et al., 1998; Huang et al., 2012; Wu et al., 2015; Zedan and AlJaber, 2019). The presence of abundant oxygen vacancies promotes the mobility of lattice oxygen and subsequently boosts the catalytic activity of CeO_2_. Meanwhile, there may also exist a synergistic effect between the doped metals and Ce. Palella *et al.* recently prepared MnCeO_x_ composites *via* a redox route and demonstrated that Mn^2+^ ions are the active sites for the formation of active oxygen species arising from the oxygen vacancies in low-temperature oxidation of CH_4_, while Ce elements can improve the availability of reactant molecules at the active site by introducing oxygen vacancies (Palella et al., 2021). There is a strong synergistic effect between Mn–Ce elements, which stabilizes Mn^2+^ species and facilitates the dispersion of Mn^2+^ ions. The dissociation of CH_4_ into CH_n_* and H* species was found to be the rate-determining step for the Mn-doped CeO_2_ composite system. Apart from Mn, other monometallic elements such as Cu (T_50_ = 350°C), Fe (T_50_ = 378°C), Ni (T_50_ = 415°C), and Co (T_50_ = 380°C) catalysts have also improved catalytic activities and stabilities due to the established synergistic effects that increase the dispersion of active metal oxides, which allow more M^n+^/M^(n−1)+^ redox couples to participate in the redox cycle; more discussions on the doped CeO_2_ catalysts can be found in a recent review by Stoian *et al.* in details (Stoian et al., 2021). Due to the absence of active site, which triggered by grain growth, CeO_2_ is sintering at high temperature. Larrondo *et al.* tried to increase the thermal stability of CeO_2_ by the addition of ZrO_2_ to the structure of CeO_2_ and found that ZrO_2_ can also slightly facilitate the reducibility of the solids associated with both surface and bulk Ce sites with the evidence of H_2_ TPR. And the catalysts with 10% of Zr have higher values of CH_4_ conversion than the other samples with 30% Zr and 50% Zr (Larrondo et al., 2005). Recently, Toscania *et al.* used the citrate complexation route to future prepared Ce_0.9_Sc_x_Zr_0.1−x_O_2−δ_ (x = 0, 0.02, 0.04, and 0.06) by citrate complexation route, with the aim of combining the improved thermal stability provided by the ZrO_2_ with an increase in vacancy concentration upon Sc doping (Toscani et al., 2019). The doped samples exhibited superior redox behavior because the CeO_2_ reduction values from TPR experiments and vacancy concentration from Raman tests both increase with increasing Sc content. And in contrast to the binary CeO_2_–ZrO_2_ sample, the CeO_2_–ZrO_2_–Sc_2_O_3_ showed the higher reaction rates and lower apparent activation energies for CMC. In addition, *in situ* XANES experiments confirm the participation of the lattice in the redox mechanism. Huang *et al.* prepared a series of NiO/CeO_2_ by a facile impregnation method, which exhibited high catalytic performance and stability due to synergistic interaction between CeO_2_ and NiO. The incorporation of Ni^2+^ into the CeO_2_ lattice obviously enhanced the concentration of oxygen vacancies and amount of surface oxygen, making the mobility of bulk oxygen in CeO_2_ increased, along with the reduction of the activation energy of the CMC. On the other hand, CeO_2_ prevented the aggregation of NiO, further improving the reduction properties of NiO (Huang et al., 2020). Apart from this, CeO_2_-modified catalytic materials can also be used in the reaction of oxidative coupling of methane (OCM) because of its above redox properties and increased surface basicity (Siakavelas et al., 2021). OCM is an exothermic reaction between CH_4_ and O_2_ in the range of 700–900°C, forming C_2_ hydrocarbons (e.g., C_2_H_4_ or C_2_H_6_), *via* CH_4_–CH_3_–C_2_H_6_–C_2_H_4_ progress. Moreover, for OCM, the incorporation of *f*-block elements such as Pr^3+^, Sm^3+^, and La^3+^ (redox-active basic ions) into the CeO_2_ could modify the acid–base properties, enhance its thermal stability, and generate additional oxygen vacancy sites (Xu et al., 2019; Zhang et al., 2020; Siakavelas et al., 2022a). Furthermore, Siakavelas et al. (2022b) recently added lithium ions into CeO_2_- and CeO_2_-modified materials (Sm-Ce and La-Sm-Ce metal oxides), using the wet impregnation technique. They argued that the addition of lithium species changed the reaction pathway and drastically enhanced the production of ethylene and ethane, mainly for the promoted catalysts (Li/Sm-Ce and Li/La-Sm-Ce).

#### 3.6.3 Binary CeO_2_ catalysts

**FIGURE 10:**
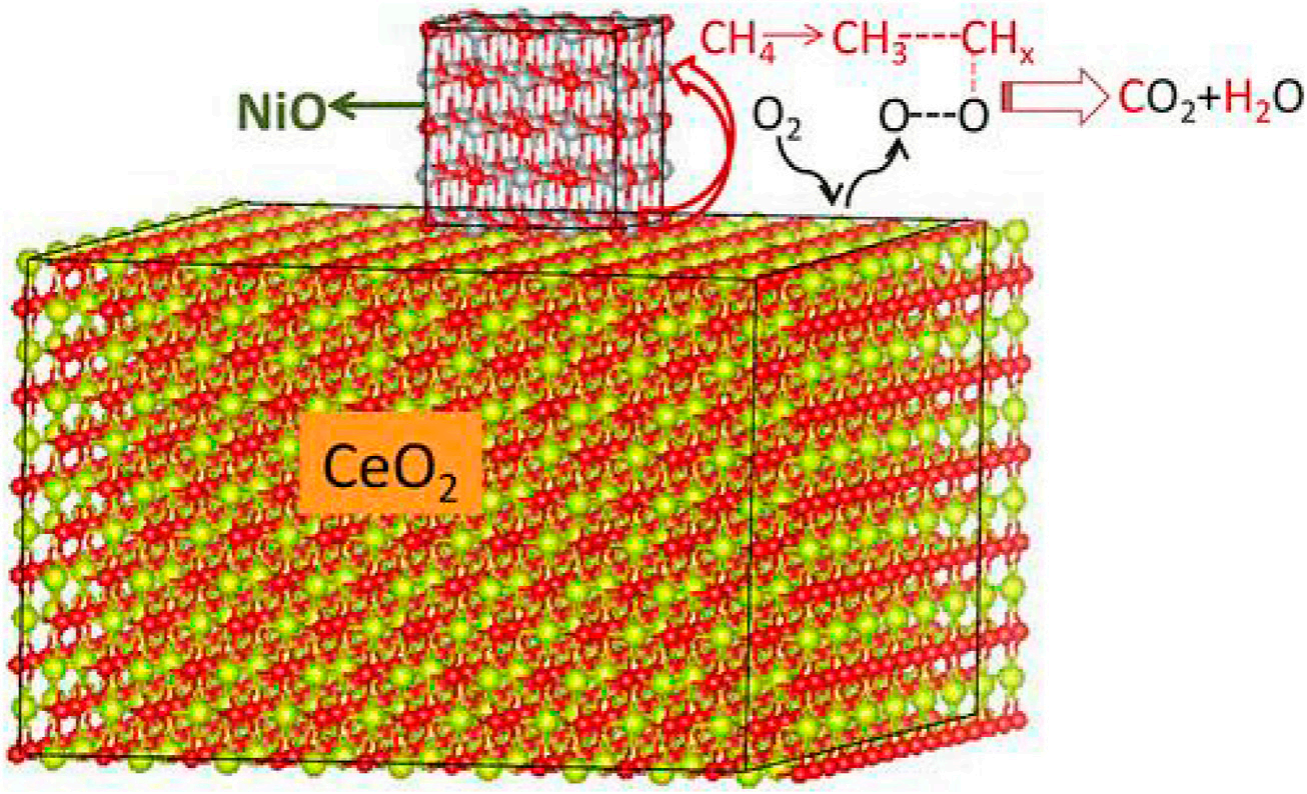
Schematic briefly showing activations of C–H of CH_4_ and O−O of O_2_ and coupling between CH_x_ species and atomic oxygen at the interface of NiO/CeO_2_. Reprinted with permission from Zhang et al. (2018). Copyright (2018) American Chemical Society.

The CMC involves both the activation of C–H of CH_4_ and O–O of O_2_. CeO_2_ can activate molecular oxygen *via* Ce^4+^ and Ce^3+^ redox couples; however, it is incapable of activating the C–H of CH_4_. On the contrary, NiO exhibits high activity in activating C–H, but, does not activate molecular oxygen. Thus, Zhang *et al.* synthesized NiO/CeO_2_ through a two-step method, in which nanocomposite consists of CeO_2_ nanorods with supported NiO nanoclusters, exhibiting notably higher activity due to the lowest apparent activation energy (69.4 ± 4 kJ/mol). The schematic diagram of the reaction mechanism at the interface is shown in Figure 10. The C–H of CH_4_ was activated on the Ni-O species, forming a H_3_C–Ni- intermediate on the interface. The formed CH_3_ could be further activated to form CH_2_ or even CH species, which could couple with surface lattice oxygen atoms to form CO_2_ and H_2_. The process follows the MvK mechanism, in which NiO nanoclusters and CeO_2_ nanorods show a synergistic effect for CMC (Zhang et al., 2018).

#### 3.6.4 Mechanisms

In conclusion, the mechanism of CeO_2_ in CMC has been reported to follow the MvK mechanism (Knapp and Ziegler, 2008). Results reported in the literature indicate that the first step is hydrogen abstraction from the CH_4_ molecule over the Ce surface. As the (111) surface of CeO_2_ is the most stable and predominant, therefore, the exposed oxygen atoms with low coordination on the (111) surface are assumed as the active sites over CeO_2_-based catalysts in CMC. CH_4_ dissociation is followed by the formation of a CH_3_ radical and an adsorbed H* atom. These two species bind to two surface oxygen atoms, leading to the formation of a methyl radical (CH_3_^−^) and a hydroxyl radical (HO·). Hydrogen adsorption leads to the reduction of one of the neighboring Ce atoms. Afterward, a series of intermediate steps take place, in which the H atoms are abstracted from CH_3_* to form CH_2_* and CH* until CO is formed by further reducing the Ce with the formation of an oxygen vacancy. Finally, the adsorbed CO reduces Ce by binding with another oxygen allowing the formation of CO_2_. Water is formed by the binding of adjacent OH groups. The breaking of the C–H bond is generally the rate-limiting step in all methane activation processes (Tang et al., 2010).

### 3.7 CuO-based catalysts

#### 3.7.1 Single CuO

CuO is an important p-type semiconducting material with a cubic rock salt structure (c-CuO) and lower-symmetry monoclinic structure (m-CuO). Analysis of the calculated band structures revealed that c-CuO is an indirect gap semiconductor, while m-CuO has metallic behavior (Cao et al., 2018). CuO has been considered as one of the most effective alternatives to noble metal-based catalysts in CMC because of its Earth abundance, non-toxicity, and good catalytic performance (Liu et al., 2010).

Researchers exhibited that Cu loading and the nature of the carrier exert influence on the Cu species present on the catalyst surface. Park and Ledford tested the catalytic activity of Cu/Al_2_O_3_ catalysts with different Cu loadings for CH_4_ combustion reactions and they found that, with increasing Cu content, both the activity per unit mass of Cu and per mole of Cu on the surface decreased (Park and Ledford, 1998). The active phase for CH_4_ oxidation is a superficial phase formed by isolated or highly dispersed Cu; as the Cu content increases, the dispersion becomes worse and therefore reduces the total number of active sites. Aguila *et al.* investigated CuO-loaded catalysts with porous media such as Al_2_O_3_, ZrO_2,_ and SiO_2_ prepared by the impregnation method (Aguila et al., 2008). This work showed that CuO catalysts supported on ZrO_2_ have higher activity (per unit mass of Cu) for CH_4_ oxidation than when CuO is supported on alumina or silica, which is related to the ability of ZrO_2_, stabilizing the highly dispersed Cu species to prevent the formation of bulk CuO. This ability is available when Cu concentrations between 0.25% and 6% for the catalysts are supported on ZrO_2_. In addition, they also tested the influence of water for the CuO catalysts supported on ZrO_2._ The addition of water produces a decrease of the CH_4_ conversion, but, as soon as the water flow is stopped, the catalyst recovers its initial activity, which means that inhibition with water is reversible, at least for the 300 min considered in this experiment.

In addition to dispersion, the acid–base properties of the supports also affect the catalytic performance of Cu-based catalysts. Theoretically, the adsorption and activation of hydrocarbons on oxide-based catalysts and the desorption of reaction products are related not only to the strength and distribution of the Lewis acidic metal cation sites but also to the concentration of lattice oxygen anions as the Lewis base sites (Vedrine et al., 1996). Specifically, the interaction of CH_4_ with acid–base pairs on the catalyst surface leads to the heterolytic breakage of the C–H bond and the formation of CH_3_^−^ and H^+^ species chemisorbed on the acid and base sites, respectively. Stronger acidic sites enhance the interaction with the carbon anion and therefore facilitate surface catalytic combustion (Choudhary and Rane, 1991). By co-precipitation and calcination methods, Popescu *et al.* synthesized the CuO nanoparticles supported on mixed oxides of Al_2_O_3_, MgO, and Mg(Al)O and investigated their catalytic properties in the total oxidation of CH_4_ (Popescu et al., 2017). Because, on the one hand, CH_4_ activation involving the heterolytic C–H bond breaking needs acid–base pairs, on the other hand, the total oxidation reaction is favored in the presence of acid sites of high strengths, which strongly adsorb carbanions, thus undergoing surface reaction with oxygen. The CuMgAl(1)O (Mg/Al atomic ratios is 1) showed the highest activity as it not only contained strong acid sites (ca.50%), which was similar with the CuAlO (strong acid species), but also had strong basic sites (ca. 30%).

#### 3.7.2 Doped CuO

**FIGURE 11:**
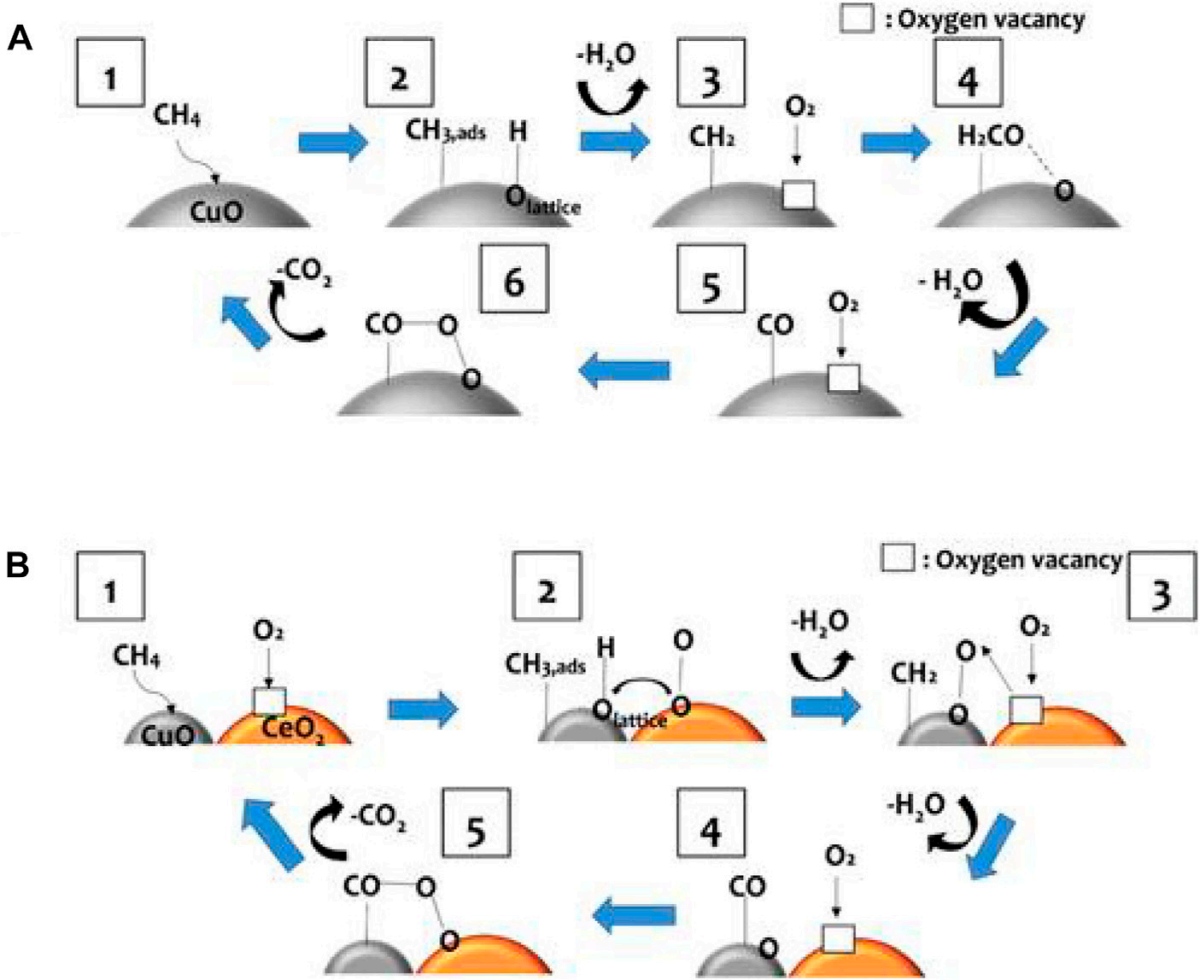
Cartoon depiction of methane combustion catalyzed by CuO **(A)** and Cu/CeO_2_ **(B)**. Reprinted with permission from Lu et al. (2016). Copyright (2016) American Chemical Society.

Lu *et al.* prepared CuO–CeO_2_ hybrid nanoparticle and created substantial amounts of Cu–Ce–O interfaces by gas-phase evaporation-induced self-assembly. CuO–CeO_2_ exhibited excellent catalytic performances with a low light-off temperature, high activity, selectivity, and operation stability. The two possible mechanisms of CMC over CuO and CuO–CeO_2_ are shown in Figure 11. The authors conjectured that two routes follow the MvK mechanism. However, the Cu–Ce–O interfacial metal–support interaction promotes the redox cycle of interface, in which CH_4_ binds to the surface of CuO, while O_2_ is simultaneously adsorbed on the oxygen vacancy of CeO_2_, then dissociating to a surface-bound methyl group and oxygen atoms, respectively; after the release of H_2_O, an oxygen vacancy regenerates, subsequently combining with the oxygen atoms; the dissociated methyl group is further oxidized by the adsorbed oxygen atoms, and then CO_2_ is released from the surface of CuO. The synergistic effect between CuO and CeO_2_ increases the rate of CMC, which has shown promise in enhancing the removal rate of hydrocarbon from the catalyst surface (Lu et al., 2016).

#### 3.7.3 Mechanisms

**FIGURE 12:**
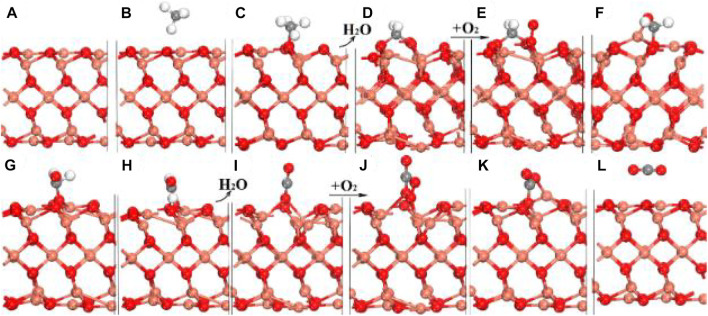
**(A)** Clean (001); **(B)** CH_4_ physical adsorption; **(C)** CH_4_ dissociative adsorption; **(D)** CH_2_* with oxygen vacancy (OV) presented; **(E)** CH_2_* with O_2_ adsorbed; **(F)** CH_2_* rotating and interacting with O_2_*; **(G)** transition state (TS) state COH_2_*; **(H)** CHO* + H*; **(I)** CO* with OV presented after releasing one H_2_O; **(J)** CO* with O_2_ adsorbed; **(K)** TS for O transfer to CO*; **(L)** CO_2_ physical adsorbed. Cu, O, C, and H are shown as rose-carmine, red, grey and white spheres, respectively. Reprinted with permission from Kong et al. (2018). Copyright (2018) Beilstein-Institut.

Kong *et al.* identified CuO (001) as an active surface for CMC over CuO among other surfaces, including (110), (111), (101), (010), and (011) *via* DFT calculations (Figure 12). It is not surprising because (001) is strongly polarized and shows high reactivity due to the high ratio of lowly coordinated oxygen. In DFT calculations, CH_4_ is firstly adsorbed with AE = -0.86 eV, followed by a spontaneous dissociation with CH_3_ and H adsorbed on surface oxygen, as depicted in Figures 12A–C. Surface oxygen is often actively involved and may cleave H from CH_x_ to form -OH and H_2_O, generating an oxygen vacancy (OV), which can be filled with reactant O_2_, leading to its dissociation and further oxidizing CH_x_ intermediates, as shown in Figures 12D–F. With oxygen transferring to bond with carbon, the hydrogen in CH_x_ can shift to surface oxygen again with an energy release of 3.66 and 1.46 eV, indicating that such a shift is highly favorable, as shown in Figures 12G–I. Again, an OV is generated and refilled by O_2_ when H_2_O is released, and similarly O_2_ is dissociated to release atomic oxygen after exceeding a small barrier of 0.47 eV, which can oxidize adsorbed CO to form CO_2_, as outlined in Figures 12I–L. Although the authors did not specify which mechanism the mechanism of CMC over CuO (001) conforms to, given their theoretical calculations, we believe that it is consistent with the MvK mechanism (Kong et al., 2018).

## 4 Perspectives

It has been well acknowledged that TMOs are one of the most promising candidates as alternatives to noble metal catalysts in CMC. Although promises have seen in some TMOs, problems still exist such as the controversial reaction mechanism, low water vapor, and sulfur resistance. Therefore, it is necessary to explore appropriate preparation methods, suitable modified ions, and the optimal ratios to synthesize novel TMOs with excellent resistance to water vapor and sulfur poisoning, guided by the reaction mechanism (Lin et al., 2017; Chen et al., 2022).

Some TMOs with significant water resistance, such as NiO, should be further studied to enhance sulfur resistance. Cr_2_O_3_ with excellent sulfur resistance can be selected to prepare binary complexes to enhance its catalytic stability (Ordóñez et al., 2008). Although α-Fe_2_O_3_ catalysts, especially with two-dimensional nanostructure, have been proven to have excellent catalytic performance in CMC, their performance is significantly reduced in the presence of steam and SO_2_. Herein, we can further investigate Fe–Cr mixed catalysts with the emphasis on the creation of spinel structures to increase the sulfur and water resistance of iron-based catalysts (García-Vázquez et al., 2020).

In addition, beyond complete oxidation, TMOs also find great uses in CH_4_ partial oxidation reactions, such as direct oxidation to methanol, which is known as the “holy grail” in catalysis community to convert CH_4_ into value-added chemicals with a much reduced cost in reaction steps and separation as compared to the conventional industrial route. (Sushkevich et al., 2017). The TMOs reveal the enormous potentials in the direct oxidation of CH_4_ to CH_3_OH owing to the relatively strong ability to activate the C–H bond and avoid CH_4_ overoxidation.

In nature, CH_4_ monooxygenase can directly convert CH_4_ to CH_3_OH using H_2_O, O_2_, and CO_2_ as reactants at an ambient temperature (Sun et al., 2021). However, such biomimetic strategies are often limited by industrial-scale reactions. Some TMOs can dissociate CH_4_ at room temperature, which offers the possibility for the direct conversion of CH_4_ (Huang et al., 2021). Zuo *et al.* found that an inverse CeO_2_/Cu_2_O/Cu (111) catalyst is able to bind and dissociate CH_4_ at room temperature by mimicking the function of the CH_4_ monooxygenase. The catalytic system produced only adsorbed CH_x_ fragments in the presence of H_2_O, along with a high transformation from CH_4_ to CH_3_OH. The dissociation of H_2_O formed OH groups, which occupied the catalyst surface. OH groups removed sites decomposing CH_x_ fragments, generating centers with special electronic properties. On the special active centers, CH_4_ could directly interact to yield CH_3_OH (Zuo et al., 2016). Liu *et al.* carefully studied key roles of H_2_O for the conversion of CH_4_ directly into CH_3_OH on CeO_2_/Cu_2_O/Cu (111). H_2_O preferentially dissociated over the active Ce sites at the CeO_2_/Cu_2_O/Cu (111) interface, hindering O_2_ activation and the overoxidation of CH_4._ H_2_O produced active *OH to promote the direct conversion of CH_4_. O_2_ dominantly reoxidized the reduced CeO_x_, and water adsorption also displaced the produced methanol into the gas phase (Liu et al., 2020).

Some researchers have explored bimetallic oxides with dual roles, in which one oxide activates CH_4_ and the other ensures high selectivity to methanol. Yang *et al.* synthesized highly mixed hybrid IrO_2_/CuO *via* a bottom-up tactic, which exhibited excellent catalytic performance with a methanol yield of 1937 μmol gcat^−1^ and a methanol selectivity of about 95% through the synergistic effect of IrO_2_ for CH_4_ activation and CuO for selective oxidation. In the oxidation process of methane, due to the strong electrophilic property of Ir^4+^, IrO_2_ could facilitate the C–H bond cleavage by forming Ir-C σ bond. Then, -CH_3_ attached by Ir bound to the neighboring Cu-attached O to form -OCH_3_, subsequently extracted by H_2_O to accelerate the formation of methanol. At last, the formed O vacancy is replenished by O_2_ (Yang et al., 2019).

It deserves to explore novel metal oxides with low metal-O bond strength and satisfactory methanol selectivity for facilitating the surface methoxy group formation. The metal oxides can also be employed as a cocatalyst being strong electrophilic metal oxides with extraordinary capacity to promote the C–H bond cleavage of CH_4_ (Fuller et al., 2016). The synergistic effect of bimetallic oxides offers an alternative route for the design and synthesis of novel catalysts for the direct conversion of CH_4_ into methanol (Lyu et al., 2021). In addition, the mechanism by which H_2_O promotes the high selectivity of direct CH_4_ conversion also needs to be continuously explored.

## 5 Conclusion

As an important greenhouse gas, the lean emission of CH_4_ causes a huge environmental crisis. In this article, all the described results certify that both single and binary TMOs show the great potential of being promising alternatives to the expensive noble metal catalysts. Reviewed novel TMO catalysts exhibit appreciable catalytic reactivity associated with preparation methods, structures, morphologies, exposed crystal planes, crystal defects, oxygen vacancies, doping, and supporting. It can be seen that the structure, morphology, and exposed crystal planes determined by the preparation method significantly influence their catalytic activity for the CMC through the variation in morphology, surface area, and surface or lattice defects. In addition, mixed TMO catalysts prepared by doping and supporting exhibit excellent catalytic performance compared with the corresponding single TMOs due to synergistic interactions between the different TMO species. In general, the deactivation of TMO catalysts due to water vapor poisoning is reversible, while the deactivation due to sulfur poisoning is irreversible. The doping and supporting will improve the stability of TMO catalysts. The reaction mechanism of CMC over TMO catalysts is still controversial. Among the discussed possibilities, the MvK mechanism involves the oxidation of methane only by lattice oxygen with molecular oxygen replenishing the lattice oxygen after its consumption, while the L–H and E–R mechanisms only involve surface-adsorbed oxygen from molecular oxygen in the gas phase as the oxidizing power for methane activation. In addition, the T-T mechanism is also proposed for reactions involving both surface-adsorbed oxygen and lattice oxygen. The dominance of surface-adsorbed oxygen and lattice oxygen may change with the catalyst structure and temperature. As the characteristic techniques are under progressive development, it is expected to reveal the precise reaction mechanism of CMC over different TMO catalytic systems and therefore the identification of the true active site structures.
